# Revealing the respiratory system of the coffee berry borer (*Hypothenemus hampei*; Coleoptera: Curculionidae: Scolytinae) using micro-computed tomography

**DOI:** 10.1038/s41598-019-54157-3

**Published:** 2019-11-28

**Authors:** Javier Alba-Tercedor, Ignacio Alba-Alejandre, Fernando E. Vega

**Affiliations:** 1 Department of Zoology, Faculty of Sciences, University of Granada, Campus de Fuentenueva, 18071 Granada, Spain; 2 Sustainable Perennial Crops Laboratory, United States Department of Agriculture, Agricultural Research Service, Beltsville, MD 20705 USA

**Keywords:** Biological techniques, Zoology

## Abstract

The coffee berry borer (*Hypothenemus hampei*) is the most economically important insect pest of coffee globally. Micro-computed tomography (micro-CT) was used to reconstruct the respiratory system of this species for the first time; this is the smallest insect (ca. 2 mm long) for which this has been done to date. Anatomical details of the spiracles and tracheal tubes are described, images presented, and new terms introduced. The total volume and the relationship between tracheal lumen diameter, length and volume are also presented. The total length of the tracheal tubes are seventy times the length of the entire animal. Videos and a 3D model for use with mobile devices are included as supplementary information; these could be useful for future research and for teaching insect anatomy to students and the public in general.

## Introduction

Insects are one of the most successful lineages of animals on Earth, especially in terrestrial environments. They developed together (but independently) with myriapods and arachnids and have a unique respiratory system for gas exchange that is formed by a complex network of tubes (tracheae), branching progressively to form increasingly narrow tubes that can supply every cell in every organ; those narrower than 1 µm in diameter are known as tracheoles. Structurally, the tracheal respiratory system is an aero-vascular system that is connected to the outside world by external openings known as spiracles. Gas exchange occurs passively by diffusion, although greater ventilation can be achieved by flexing of the musculature to compress air-sacs (dilated tracheal tubes) and even the tracheal trunks themselves^1–19^. These tubes were first observed and drawn in the 17^th^ century by the Italian anatomist Marcello Malpighi in his description of silk worm anatomy^20,21^.

To enable the visualization of the tracheal tubular system, different media have been used to clear, stain, and fix specimens. Many papers report injection of the tracheal tubes with glycerol, sudan II in oil, prussian blue, trypan blue, carmine in metagelatin, osmium tetroxide vapour, silver, olive oil in petroleum, and many others. Some of these compounds require the soft tissues to subsequently be dissolved by using a special apparatus, as described by Wigglesworth^22^.

The classical methodologies used to study the tracheal system require labour-intensive dissection and highly skilled microscopic preparations. Furthermore, the specimens are destroyed in the process. In fact, recently, intact live specimens, or insect body parts, have been placed directly into lactic acid which progressively cleared the tracheal systems until the structures could be visualised; previous methods resulted in almost totally transparent specimens that were not suitable for subsequent observations^23^.

Studies on the tracheal systems of various insects published in the early-mid 20^th^ century have been covered in several reviews^1,2,15,17,18^. Detailed studies on the structure and typologies of insect spiracles have also been published^4,8,15–17,24–27^, and recently the first thoracic spiracle of the coffee berry borer (*Hypothenemus hampei* (Ferrari); Coleoptera: Curculionidae: Scolytinae) was described^28^. A few studies on the coleopteran tracheal system have been conducted using classical methods (i.e., staining and/or dissection)^16,24,29,30^. X-ray techniques were valuable in demonstrating forced respiration in beetles by compressive movements of the tracheal tubes^3,7,31^, and also in quantifying aspects of tracheal hypermetry in grasshoppers^32^. Most recently, micro-computed tomography (micro-CT) has become the most important non-destructive technique useful in revealing important anatomical insights on the complex anatomy of tracheal tubular systems in Diptera^10,33^, Orthoptera^34,35^, Coleoptera^9,36,37^ and non-insect arthropods such as camel spiders (Solifugae)^38^.

The coffee berry borer is the most devastating insect pest of coffee globally and poses a threat to coffee production due to its cryptic life habit inside the coffee berry, which makes it difficult to manage^39^. We have used modern micro-CT techniques to study this species and revealed various aspects of its biology inside coffee berries^40^, and the internal anatomy in adults^41^. In this paper, we present results from a detailed anatomical study of the tubular respiratory system of the coffee berry borer, including its spiracles. The majority of the study relies on micro-CT although additional light microscopic images are included.

## Results

**Figure 1:**
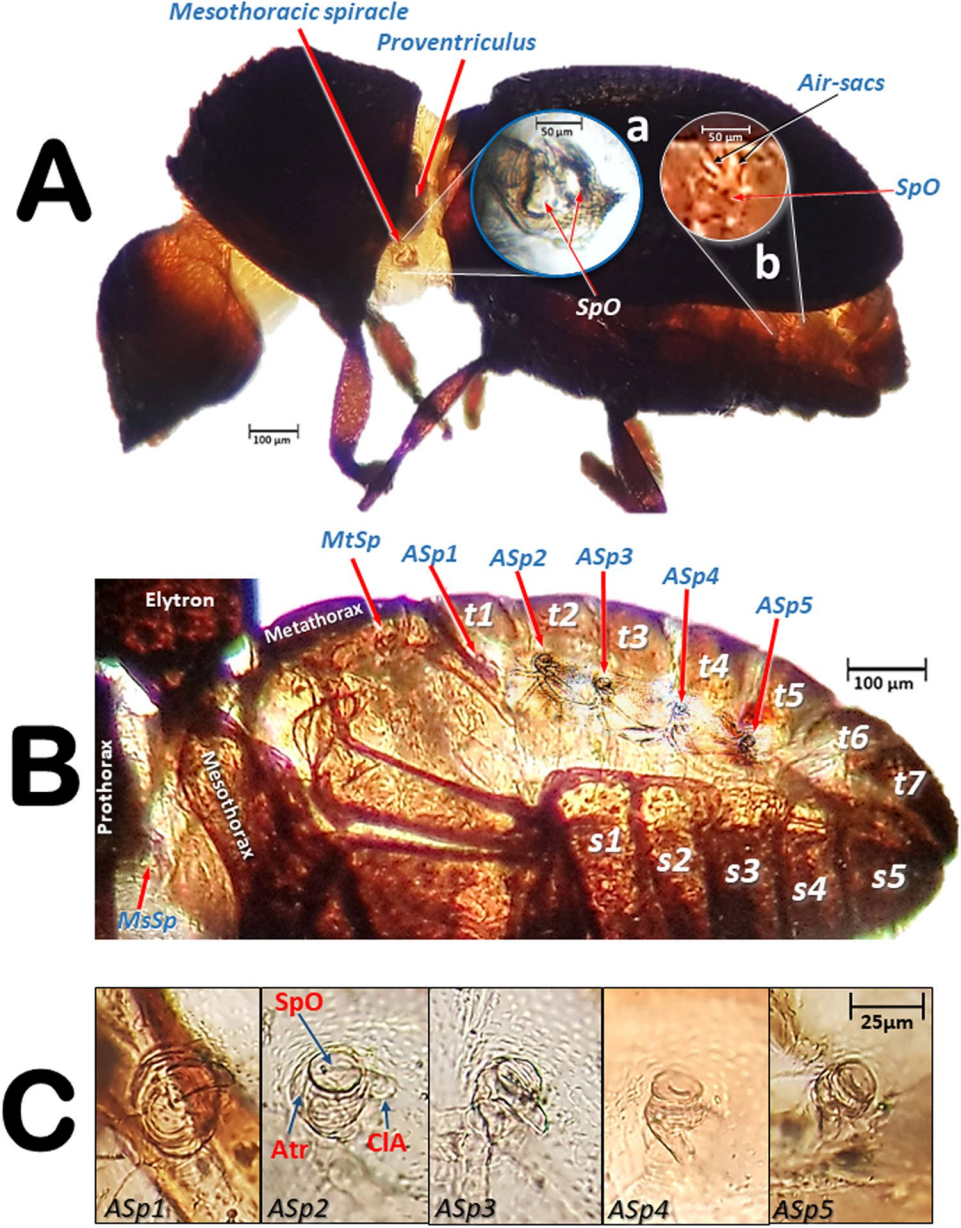
Lateral view of an adult female coffee berry borer under a stereoscope (**A**, except **A:** a), and under light microscopy *(***A**: a; **B**; **C**) after immersion in 10% KOH for 24 (**B**) and 48 h (**C**). Details of the mesothoracic (**A:** a) and 5^th^ abdominal spiracle (**A:** b). Abbreviations: ASp = abdominal spiracle; Atr = spiracular atrium; ClA = elastic bar of the closing apparatus; MsSp = mesothoracic spiracle; MtSp = metathoracic spiracle; s = sternite; SpO = spiracular opening; t = tergite.

**Figure 2:**
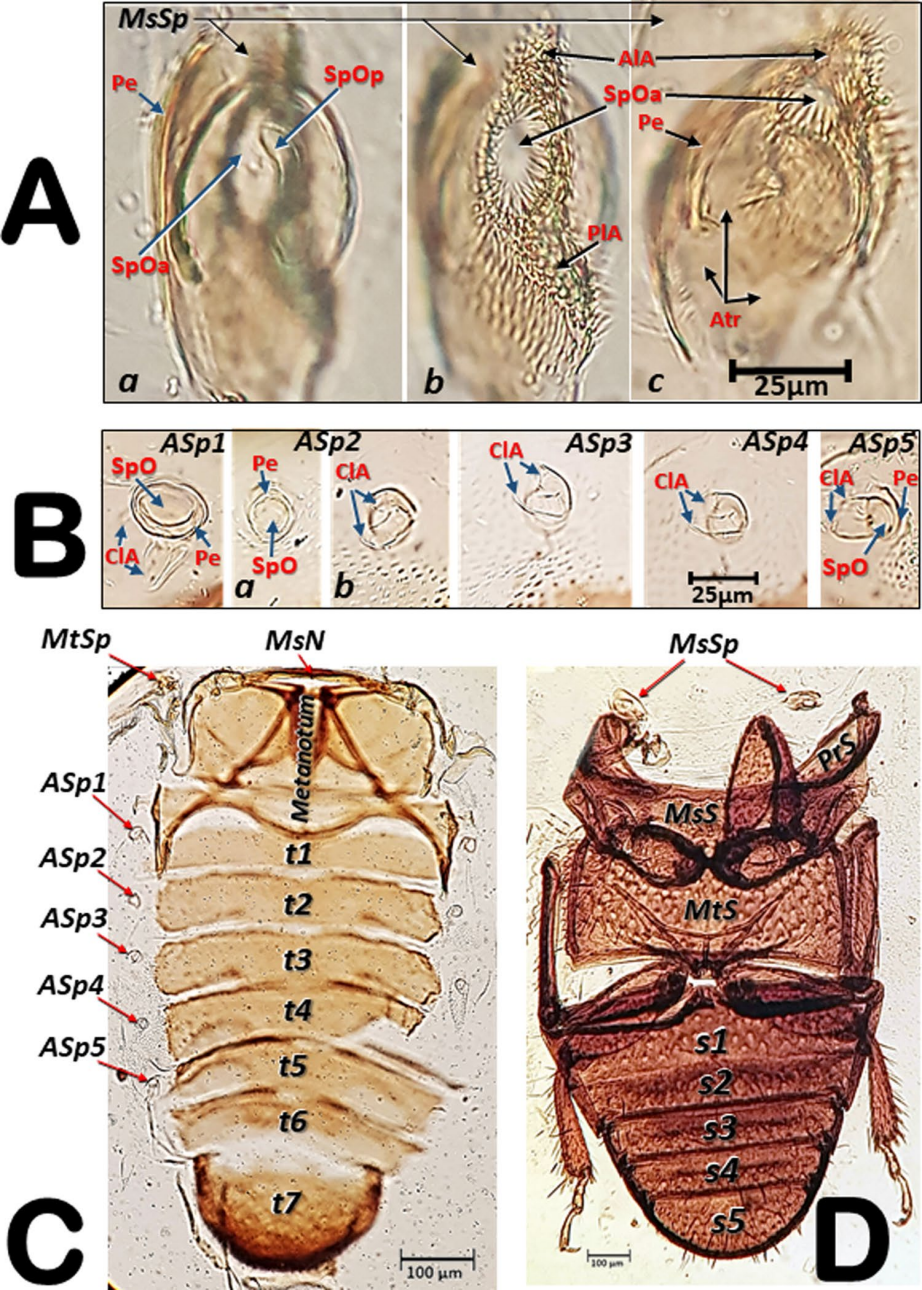
Light microscopy images of a slide preparation of an adult female coffee berry borer after clarifying in KOH for 48 h and mounting in Hoyer’s medium, showing details of the spiracles (**A**,**B**), with dorsal (**C**) and ventral (**D**) sections separately mounted. Abbreviations: AlA = anterior lip of atrium; ASp = abdominal spiracle (ASp2, in external (a) and internal (b) focused views); Atr = spiracular atrium; ClA = elastic bar of the closing apparatus; MsN = mesonotum; MsSp = mesothoracic spiracle (a: apical inner view; b: apico-external view; c: lateral view); MtSp = metathoracic spiracle; MsS = mesosternite; MtS = metasternite; Pe = peritreme; PlA = posterior lip of atrium; PrS = prosternum; s = sternite; SpOa = anterior spiracular opening; SpOp = posterior spiracular opening; t = tergite.

The external appearance of an adult female coffee berry borer with details of the spiracles obtained using light microscopy is shown in Figs. 1 and 2. As a result of the transparency achieved, in lateral view it is possible to observe a detail of the proventriculus and the mesothoracic spiracle (MsSP; Fig. 1) with two spiracular openings (SpO), and a detail of the fifth abdominal spiracle (Fig. 1Ab) in which dilatations of the lumina of the tracheal tubes can be distinguished as they converge on the spiracle. A lateral view of the thorax (hind part of the prothorax, mesothorax, and metathorax), abdomen, location of the metathoracic (MtSp) and abdominal spiracles, abdominal tergites (t1–t7) and sternites (s1–s5) are shown in Fig. 1B. Details of the five abdominal spiracles (ASp1–ASp5) in their natural position prior to mounting are visible in Fig. 1C, including the spiracle opening (SpO), atrium (Atr), and the elastic bar of the closing apparatus (ClA).

Details of the spiracles, including slide-mounted dorsal and ventral segments are shown in Fig. 2. The mesothoracic spiracle in apical view (focused internally with the peritreme) and the two external spiracular openings can be seen in Fig. 2Aa, while the apically focused anterior spiracular opening, and the anterior and posterior lips of the atrium are visible in Fig. 2Ab. The lateral view can be seen in Fig. 2Ac. The spiracular openings and the lips are densely covered with setae (Fig. 2Ab,c). Figure 2B shows the abdominal spiracles, particularly the spiracular openings (SpO), the peritreme (Pe), and the elastic bar of the closing apparatus (ClA). The second abdominal spiracle is shown in detail in an externally focused image of the spiracular opening (SpO) and the peritreme (Pe) sclerite that surrounds it (Fig. 2Ba). The images of the third, fourth, and fifth abdominal spiracles (ASp3-ASp5; Fig. 2B) are internally focused to see the elastic bar of the closing apparatus (ClA). Figure 2C shows the dorsum of a female coffee berry borer with the location of a mesothoracic spiracle (MsSp), metathoracic spiracle (MtSp), the mesonotum (MsN), metanotum (MtN), seven tergites (t1-t7), and five abdominal spiracles (ASp1-ASp5). Figure 2D presents a ventral view with the two prothoracic spiracles (PtSp), mesosternum (MsS), metasternum (MtS), and five sternites (S1-S5).

**Figure 3:**
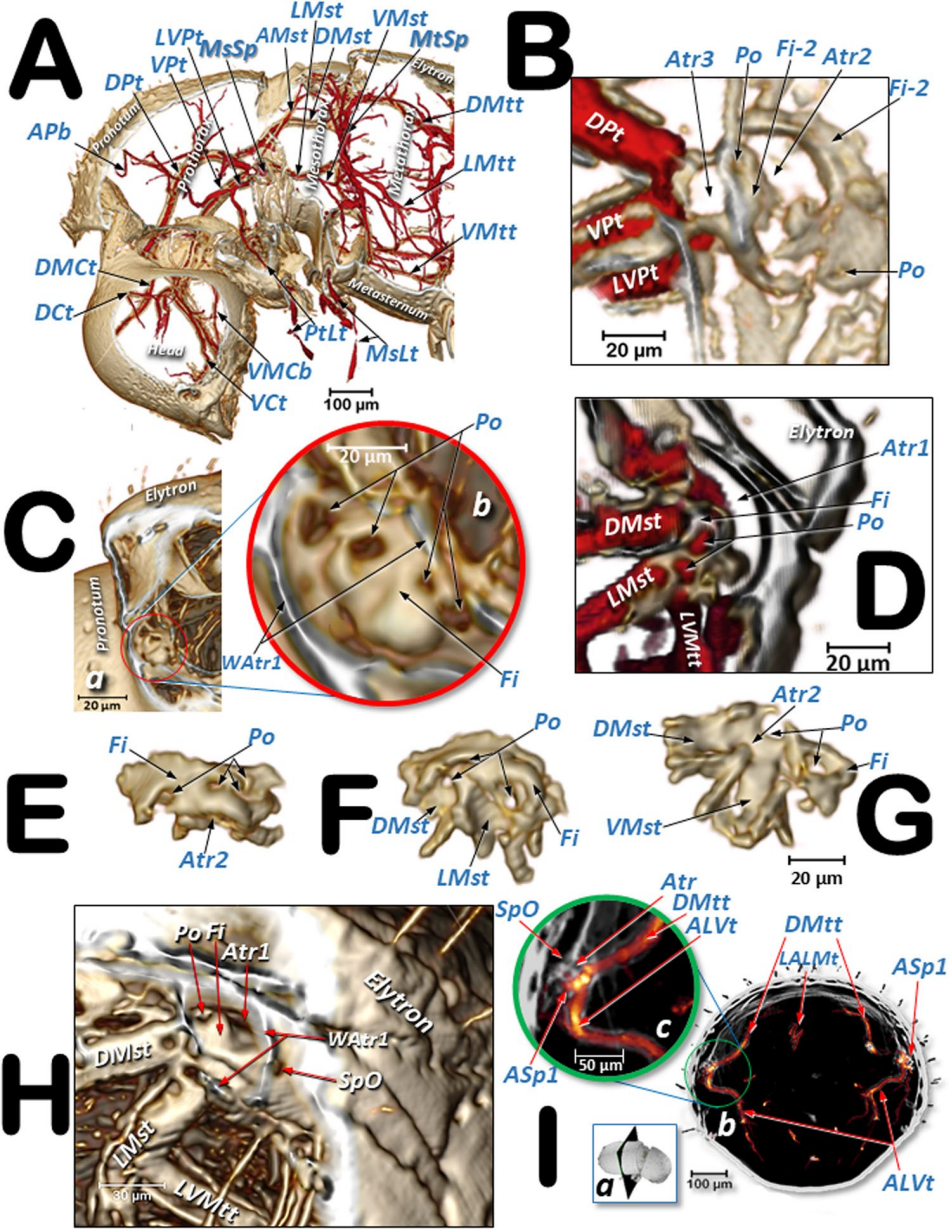
Volume-rendered images showing the location (**A**) and detailed structures (**B**–**I**) of the spiracles in relation to the tubular tracheal system. Left-sagittal slice section of the head and thorax (**A**); left-lateral slice section of the prothoracic spiracle (**B**); dorso-ventral-oblique left-lateral cut of the body to show the mesothoracic spiracle (**C**, a: in a general view; **b**: detail of the second atrial cavity opened to show the cribellated filtering structure); left-antero-posterior slice section of the mesothoracic spiracle (**D**); details of the filter in different perspectives and with the first spiracular atrium cavity sectioned, in dorso-ventral view and with two progressive rotations to provide a ventro-dorsal view (**E**–**G**); left antero-posterior cut view of the metathoracic spiracle (**H**); and Amira’s multiplanar slice section of the abdomen at the level of the first abdominal spiracle (**I**: a: position of the slice section; b: 60 µm thick slice, and c: detail of the right first abdominal spiracle). Abbreviations: ALVt = abdominal latero-ventral trunk; AMst = arc-shaped mesothoracic trunk; ASp = abdominal spiracle; Atr = spiracular atrium; DCt = dorsal cephalic trunk; DMCt = dorsal median cephalic trachea; DMst = dorsal mesothoracic trunk; DMtt = dorsal metathoracic trunk; DPt = dorsal prothoracic trunk; Fi = filter; APb = anterior prothoracic twisted branch; LALMt = long abdominal latero-median tracheae; LMst = lateral mesothoracic trunk; LMtt = lateral metathoracic trunk; LVPt = latero-ventral prothoracic trunk; MsLt = mesothoracic leg trunk; MsSp = mesothoracic spiracle; MtSp = metathoracic spiracle; Po = porus; PtLt = prothoracic leg trunk; SpO = spiracular opening; VCt = ventral cephalic trunk; VMCb = ventral median cephalic branch; VMst = ventral mesothoracic trunk; VMtt = ventral metathoracic trunk; VPt = ventral prothoracic trunk; WAtr = atrial wall.

**Figure 4:**
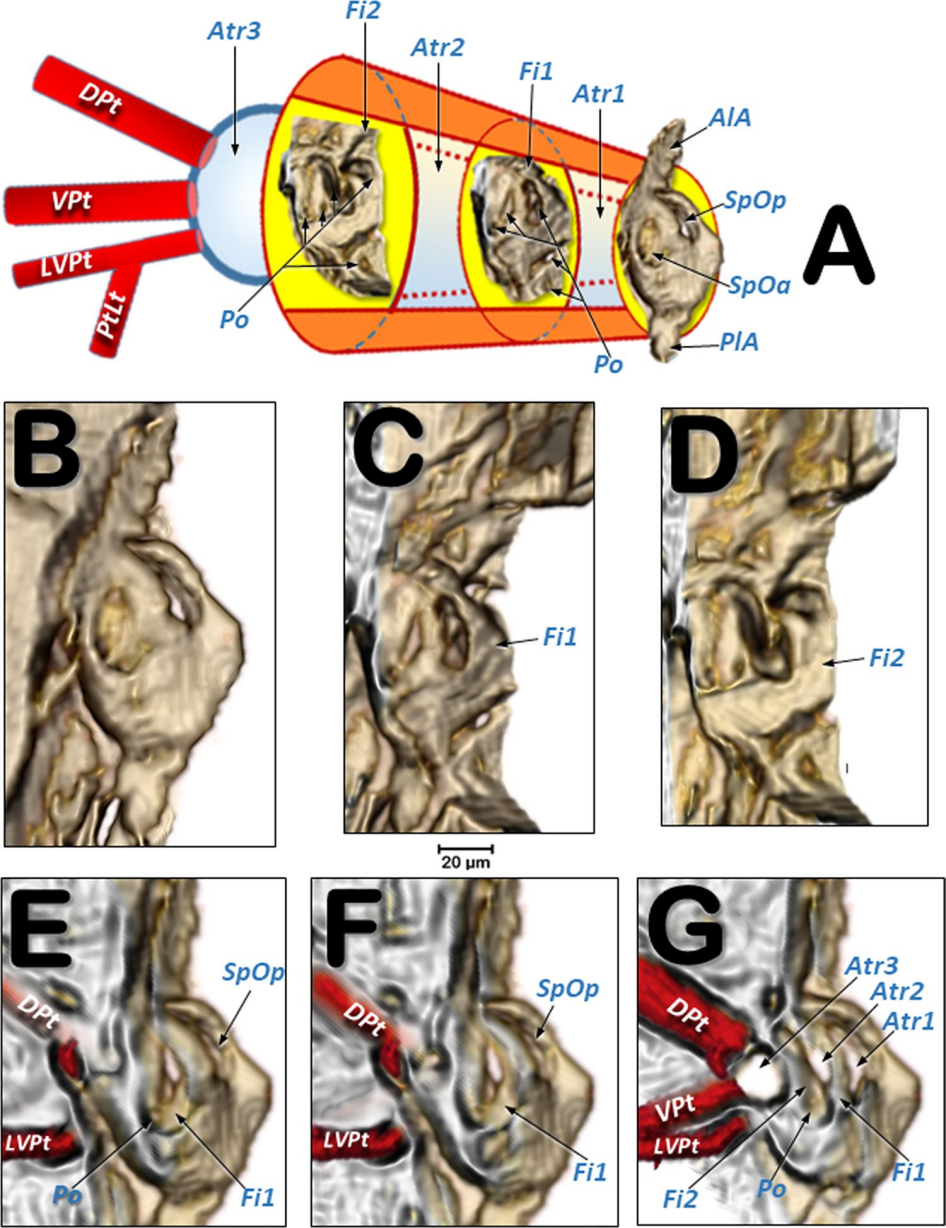
Volume rendered images of the mesothoracic spiracle (**A–G**). Schematic elongated reconstruction (**A**), external left-latero posterior view (**B**), successive virtual erosion of the external wall to show the second filter (**C**), and underneath it the first filter (**D**). Progressive virtual cuts of the left-latero posterior view shown in B, showing the tracheal tubes, atrial cavities, and filters (**E**–**G**). Abbreviations: AlA = anterior lip of atrium; Atr = spiracular atrium; DPt = dorsal prothoracic trunk; Fi = filter; LVPt = latero-ventral prothoracic trunk; Po = porus (opening); PlA = posterior lip of atrium; PtLt = prothoracic leg trunk; SpOa = anterior spiracle opening; SpOp = posterior spiracle opening; VPt = ventral prothoracic trunk.

**Figure 5:**
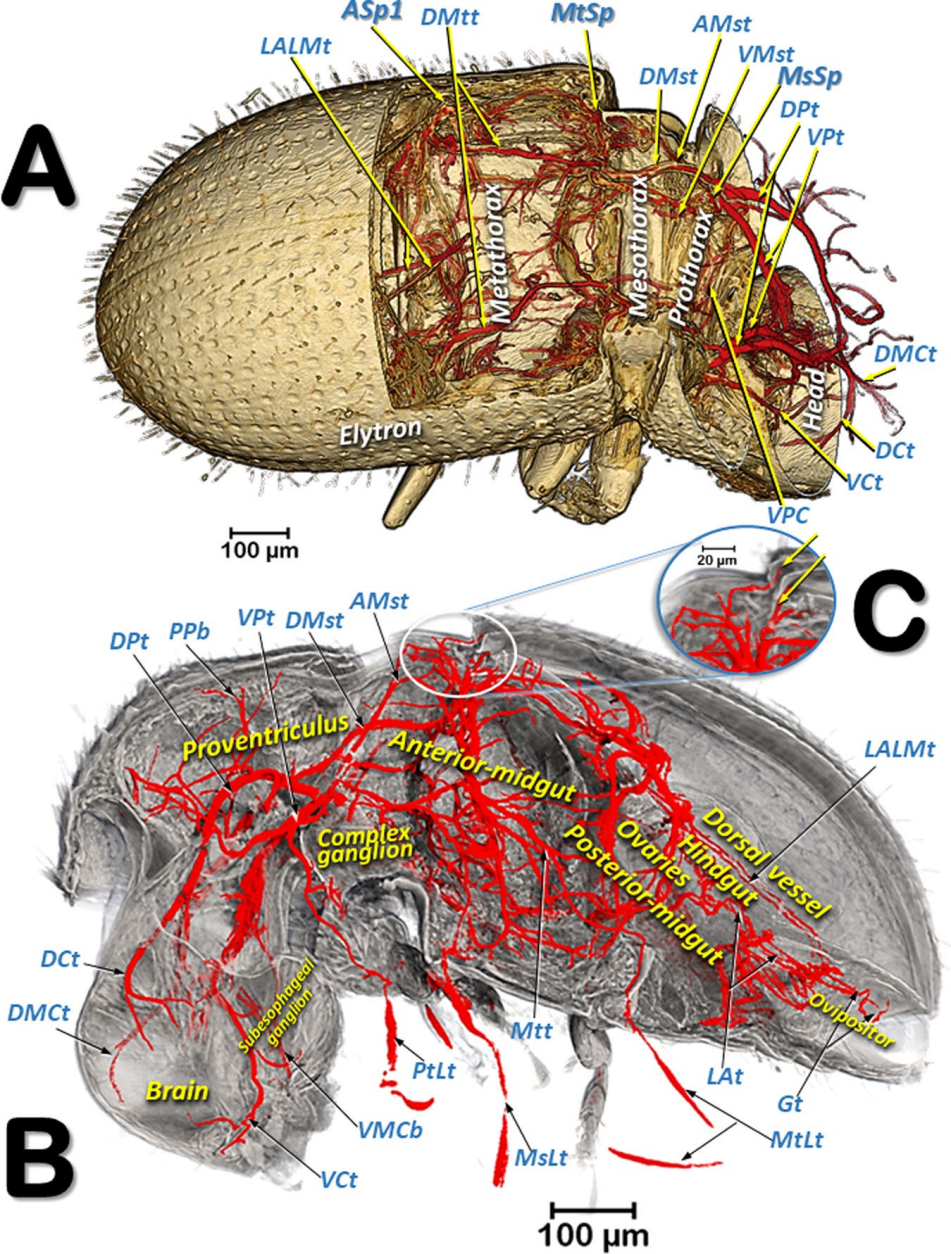
Volume-rendered images of a right dorso-lateral view where the body wall has been opened with software to show the positions of the tracheal tubes and spiracles (**A**) with a sagittal section showing the tracheal system and the position of the main internal structures (**B**), and a close-up view of the site where the tracheal tubes supply the elytron and hindwing, marked with arrows (**C**). Abbreviations: AMst = arc-shaped mesothoracic trunk; ASp = abdominal spiracle; DCt = dorsal cephalic trunk; DMCt = dorsal median cephalic trachea; DMst = dorsal mesothoracic trunk; DMtt = dorsal metathoracic trunk; DPt = dorsal prothoracic trunk; Gt = genital tracheae; LALMt = long abdominal latero-median tracheae; LAt = latero-abdominal tracheae; MsLt = mesothoracic leg trunk; MsSp = mesothoracic spiracle, MtLt = metathoracic leg trunk; MtSp = metathoracic spiracle; Mtt = metathoracic trunk; PtLt = prothoracic leg trunk; PPb = posterior prothoracic branch; VCt = ventral cephalic trunk; VMCb = ventral median cephalic branch; VMst = ventral mesothoracic trunk; VPC = ventral prothoracic commissure; VPt = ventral prothoracic trunk.

Figures 3 and 4 are micro-CT volume-rendered images showing the location and structural details of the thoracic spiracles in relation to the tracheal system. Figure 5 is a general overview of the tracheal system within the body and its relationship with the main internal anatomical structures.

**Figure 6:**
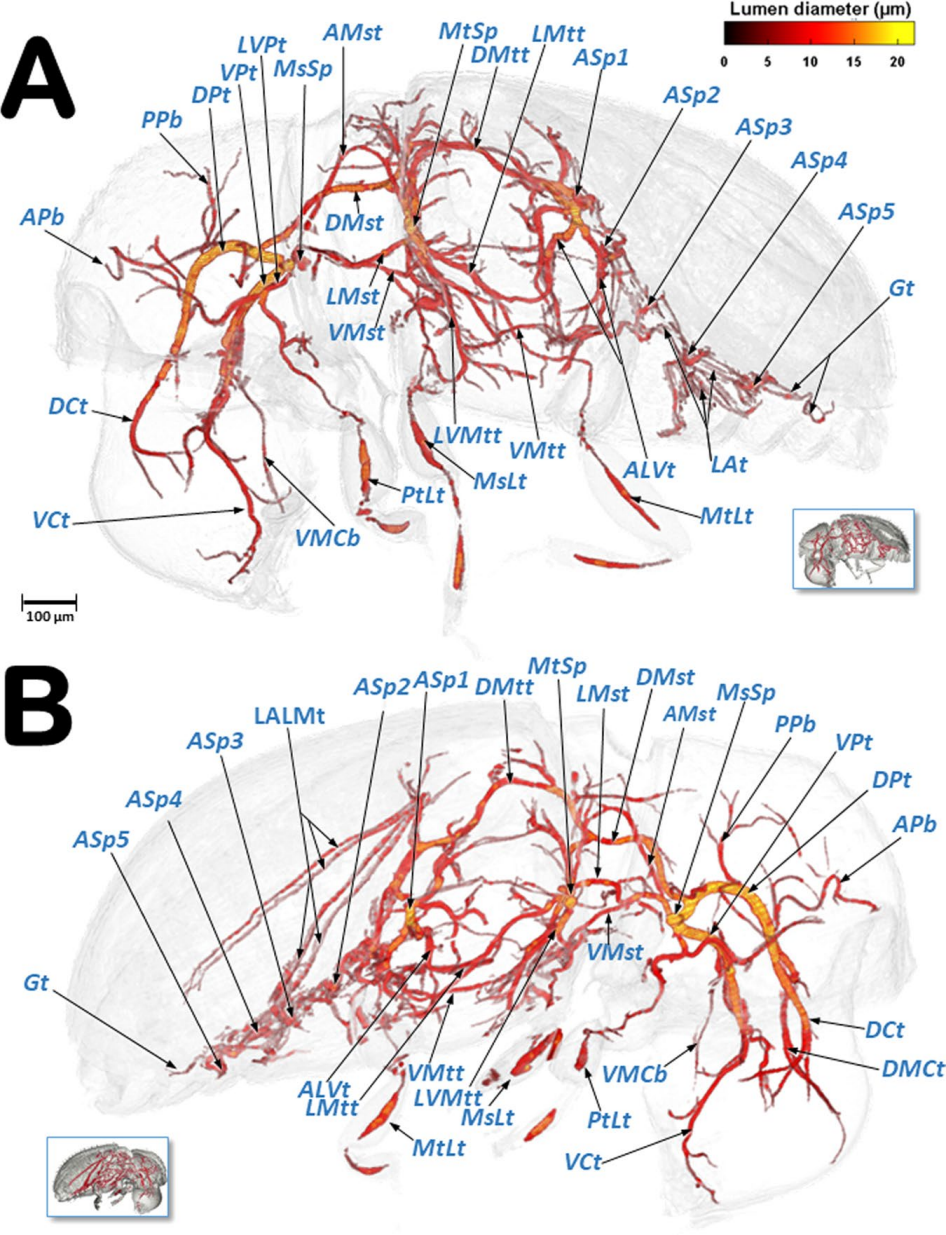
Volume-rendered images of the left (**A**) and right (**B**) lateral views of the tracheal system (the body has been made transparent). The insets correspond to volume-rendered images of the actual body position. Abbreviations: ALVt = abdominal latero-ventral trunk; AMst = arc-shaped mesothoracic trunk; APb = anterior prothoracic twisted branch; ASp = abdominal spiracle; DCt = dorsal cephalic trunk; DMCt = dorsal median cephalic tracheae; DMst = dorsal mesothoracic trunk; DMtt = dorsal metathoracic trunk; DPt = dorsal prothoracic trunk; Gt = genital tracheae; LALMt = long abdominal latero-median tracheae; LAt = latero-abdominal tracheae; LMst = lateral mesothoracic trunk; LMtt = lateral metathoracic trunk; LVMtt = latero-ventral metathoracic trunk; LVPt = latero-ventral prothoracic trunk; MsLt = mesothoracic leg trunk; MtLt = metathoracic leg trunk; MsSp = mesothoracic spiracle; MtSp = metathoracic spiracle; PPb = posterior prothoracic branch; PtLt = prothoracic leg trunk; VCt = ventral cephalic trunk; VMCb = ventral median cephalic branch; VMst = ventral mesothoracic trunk; VMtt = ventral metathoracic trunk; VPt = ventral prothoracic trunk. Lumen diameter is in accordance with the color scale of the bar shown on the upper right.

**Figure 7:**
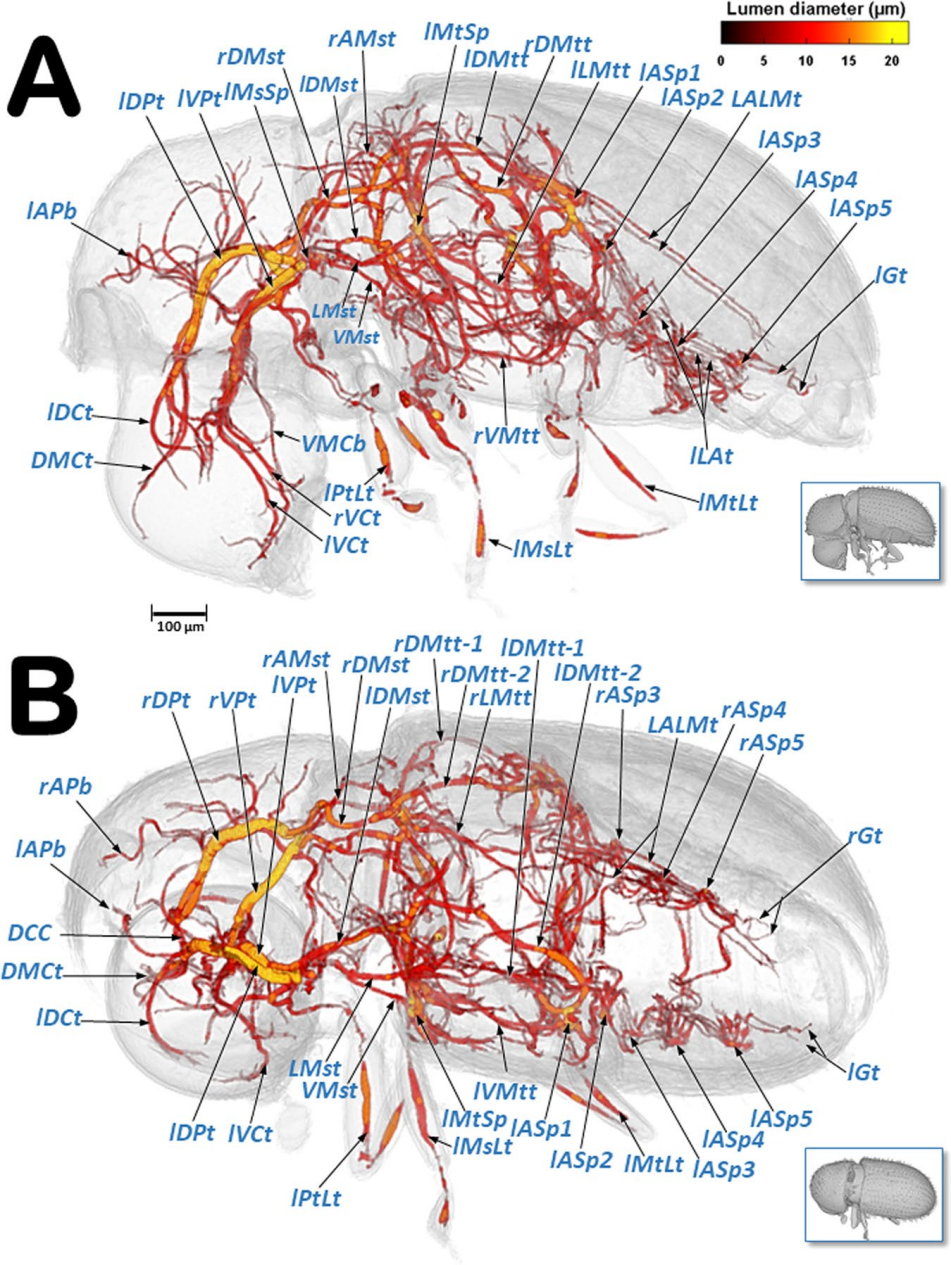
Volume-rendered images of the tracheal system in a left-lateral (**A**) and a left dorso-ventral view (**B**). The insets correspond to volume-rendered images of the actual body position. Abbreviations: AMst = arc-shaped mesothoracic trunk; APb = anterior prothoracic twisted branch; ASp = abdominal spiracle; DCC = dorsal cephalic commissure; DCt = dorsal cephalic trunk; DMCt = dorsal median cephalic tracheae; DMst = dorsal mesothoracic trunk; DMtt = dorsal metathoracic trunk; DPt = dorsal prothoracic trunk; Gt = genital tracheae; LALMt = long abdominal latero-median tracheae; LAt = latero-abdominal tracheae; LMst = lateral mesothoracic trunk; LMtt = lateral metathoracic trunk; MsLt = mesothoracic leg trunk; MtLt = metathoracic leg trunk; MsSp = mesothoracic spiracle; MtSp = metathoracic spiracle; PtLt = prothoracic leg trunk; VCt = ventral cephalic trunk; VMCb = ventral median cephalic branch; VMst = ventral mesothoracic trunk; VMtt = ventral metathoracic trunk; VPt = ventral prothoracic trunk. Right and left sides are marked with ‘r’ or ‘l’, respectively, at the beginning of each label lettering. Lumen diameter is in accordance with the color scale of the bar shown on the upper right.

**Figure 8:**
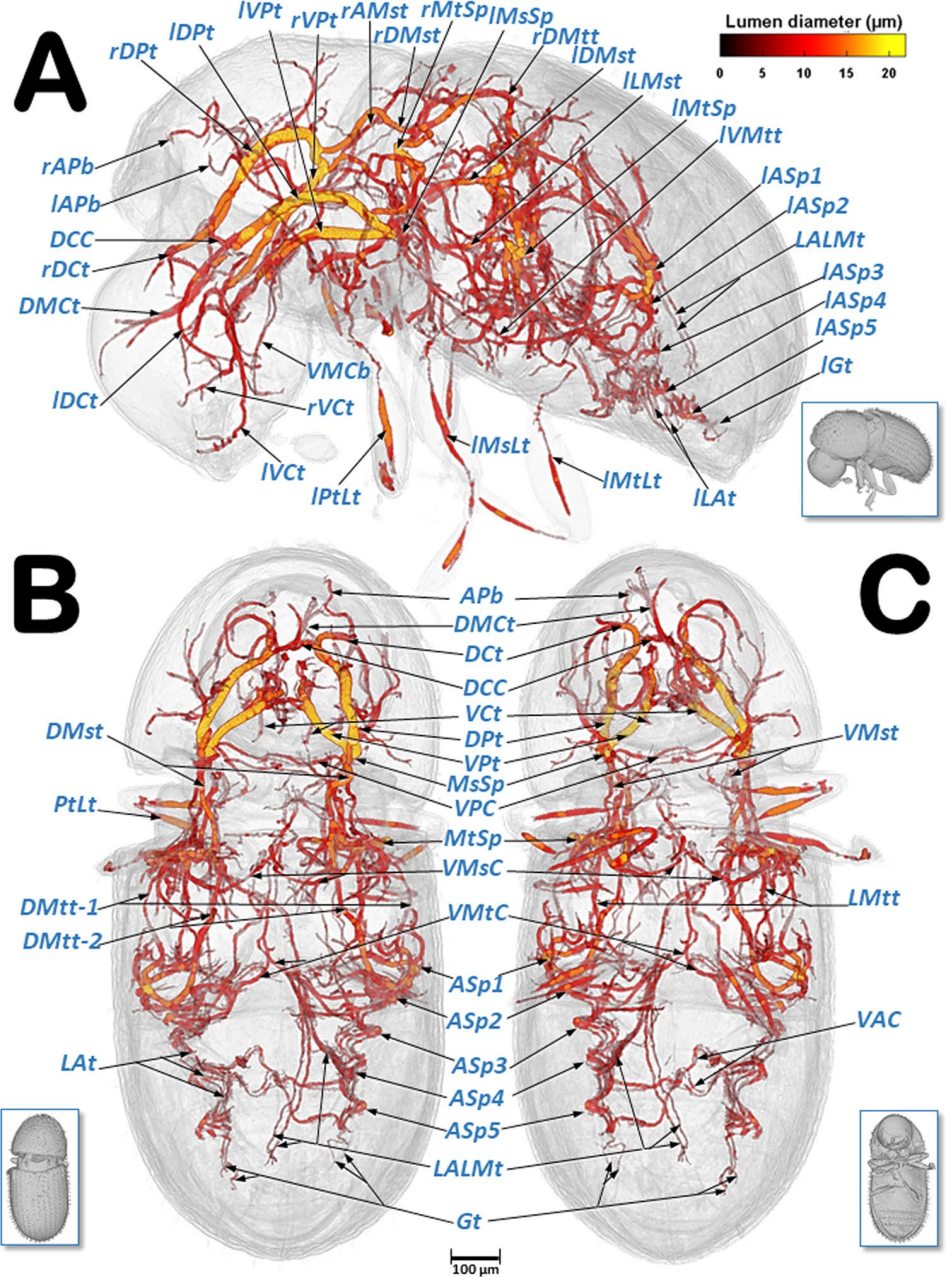
Volume-rendered images of the tracheal system in a left-lateral (**A**), dorsal (**B**), and ventral (**C**) view. The insets correspond to volume-rendered images of the actual body position. Abbreviations: AMst = arc-shaped mesothoracic trunk; APb = anterior prothoracic twisted branch; ASp = abdominal spiracle; DCC = dorsal cephalic commissure; DCt = dorsal cephalic trunk; DMCt = dorsal median cephalic tracheae; DMst = dorsal mesothoracic trunk; DMtt = dorsal metathoracic trunk; DMtt-1 = lateral branch of the dorsal metathoracic trunk; DMtt-2 = medial branch of the dorsal metathoracic trunk; DPt = dorsal prothoracic trunk; Gt = genital tracheae; LALMt = long abdominal latero-median tracheae; LAt = latero-abdominal tracheae; LMtt = lateral metathoracic trunk; MsLt = mesothoracic leg trunk; MsSp = mesothoracic spiracle; MtLt = metathoracic leg trunk; MtSp = metathoracic spiracle; PtLt = prothoracic leg trunk; VAC = ventral abdominal commissures; VCt = ventral cephalic trunk; VMCb = ventral median cephalic branch; VMsC = ventral mesothoracic commissure; VMtC = ventral metathoracic commissure; VMtt = ventral metathoracic trunk; VPC = ventral prothoracic commissure; VPt = ventral prothoracic trunk. Right and left sides are marked with ‘r’ or ‘l’, respectively, at the beginning of each label lettering. Lumen diameter is in accordance with the color scale of the bar shown on the upper right.

**Figure 9:**
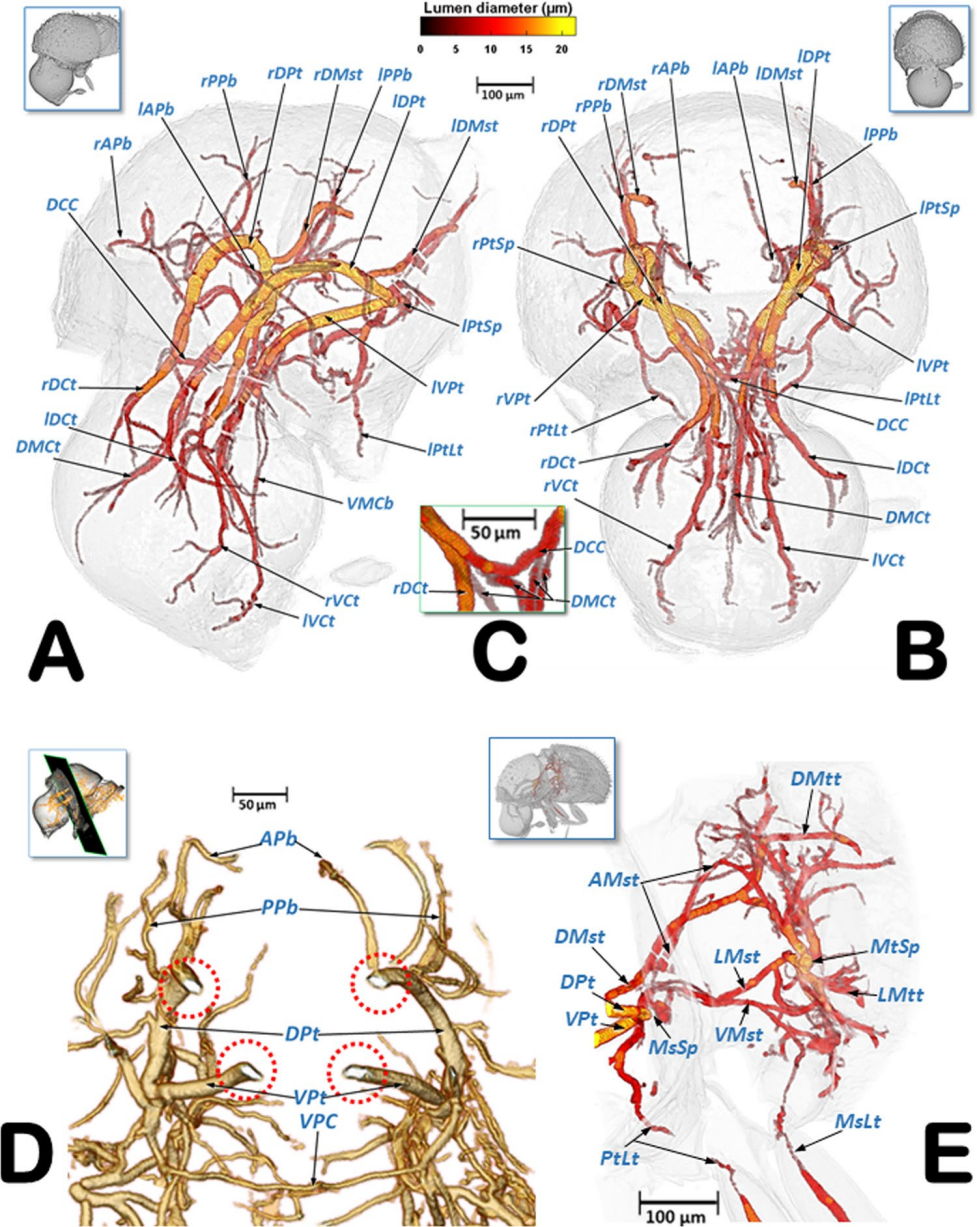
Volume-rendered images of the cephalic and prothoracic tracheal system in a left-fronto-lateral (**A**), frontal (**B**), and fronto-posterior (**D**, dotted red circles show the elliptic-shaped collapsible lumina of the tracheal trunks) view. Detail close-up of the dorsal cephalic commissure and the dorsal median cephalic tracheae (**C**), the left half of a latero-frontal view of the posterior prothorax, mesothorax and the anterior metathorax (**E**). The insets in (**A**,**B**,**E)**, correspond to volume-rendered images of the actual body position, and the one in (**D**) shows the position of the cut. Abbreviations: AMst = arc-shaped mesothoracic trunk; APb = anterior prothoracic twisted branch; DCC = dorsal cephalic commissure; DCt = dorsal cephalic trunk; DMCt = dorsal median cephalic tracheae; DMtt = dorsal metathoracic trunk; DPt = dorsal prothoracic trunk; LMSt = lateral mesothoracic trunk; LMtt = lateral metathoracic trunk; MsSp = mesothoracic spiracle; MtSp = metatoracic spiracle; PPb = posterior prothoracic branch; PtLt = prothoracic leg trunk; VCt = ventral cephalic trunk; VMCb = ventral median cephalic branch; VMst = ventral mesothoracic trunk; VPC = ventral prothoracic commissure; VPt = ventral prothoracic trunk. Right and left sides are marked with ‘r’ or ‘l’, respectively, at the beginning of each label lettering. Lumen diameter of (**A**,**B**,**E**) are in accordance with the color scale of the bar shown at the top.

Figures 6–9 show the complexity of the tubular tracheal respiratory system, viewed from different perspectives. It is important to note that many different, non-repeated angles were chosen in order to observe the positioning of the entire system. The left and right (Fig. 6B) halves are visible in sagittal cuts (Fig. 6), complemented by a left-lateral and a left dorso-ventral view (Fig. 7), and left-lateral, dorsal, and ventral views in Fig. 8. Figure 9 shows close-up views of the tracheal tubes (from the head to the anterior part of the mesothorax) in a left-frontal view (Fig. 9A) and a frontal view (Fig. 9B, and a close-up detail in Fig. 9C). A fronto-posterior view shows the elliptically shaped lumina of the tracheal trunks (Fig. 9D). The left half of a latero-frontal view of the posterior prothorax, mesothorax and anterior metathorax can be seen in Fig. 9E.

The morphological terminology used is summarized in Table 1 and where applicable we cite references that have used each term previously, other terms used with references, and new terms that we have introduced.

**Table 1:** List of abbreviations used in the paper. The references indicate studies that have previously used the same term. Alternative terms used for the same structure are listed with references, and new terms introduced in this study are noted.

Abbreviation	Name	Reference/Alternative term
AlA/PlA	anterior/posterior lip of atrium	^1^
ALVt	abdominal latero-ventral trunk	newly introduced
AMst	arc-shaped mesothoracic trunk	newly introduced
APb	anterior prothoracic twisted branch	newly introduced
ASp	abdominal spiracle	^1,7,8,17,24,25,29,30,45,55^
Atr	spiracular atrium	^1,7,17,29^/atrium of the spiracle^45^
ClA	elastic bar of the closing apparatus	^1^/closing bar^7^, occluding apparatus^8^, flexible rod compressing trachea^2^, closing bow^25^, closing apparatus^45^
DCC	dorsal cephalic commissure	dorsal tracheal commissure^1^, dorsal commissure of the head^24^, dorsal head commissure^29^, dorsal cervical anastomosis^55^, dorsal commissure^25^, brain commissure^17^
DCt	dorsal cephalic trunk	dorsal head trunk^1^, superior cephalic tracheae^24^, dorsal cervical trachea^55^, dorsal cephalic trachea^7^, dorsal head trachea^3,17^
DMCt	dorsal median cephalic trachea	newly introduced
DPt DMst DMtt	dorsal prothoracic trunk dorsal mesothoracic trunk dorsal metathoracic trunk	lateral plurisegmental tracheal trunk^1^, dorsal lateral trunk/lateral trunk^29^, large lateral longitudinal trunks^7^, lateral longitudinal trachea^30^, dorsal longitudinal trunk^17^, dorsal trachea^25^, longitudinal tracheal trunk^45^
DMtt-1	lateral branch of the dorsal metathoracic trunk	newly introduced
DMtt-2	medial branch of the dorsal metathoracic trunk	newly introduced
Fi	filter	filter apparatus^1,26,27,45^, sieve^7^
Gt	genital tracheae	^24,30^/posterior reproductive branch^29^
LALMt	long abdominal latero-median tracheae	long median trachea^29^
LAt	latero-abdominal tracheae	large lateral longitudinal trunks^7^, abdominal dorsal longitudinal trunk^17^
LMst	lateral mesothoracic trunk	newly introduced
LMtt	lateral metathoracic trunk	newly introduced
LVMtt	latero-ventral metathoracic trunk	newly introduced
LVPt	latero-ventral prothoracic trunk	ventral lateral trunk^29^, lateral longitudinal trunk^55^
MsLt	mesothoracic leg trunk	mesothoracic leg tracheae^24^, mesoleg trachea^17^
MsSp	mesothoracic spiracle	^1,29^/anterior thoracic spiracle^7,29,55^
MtLt	metathoracic leg trunk	metathoracic leg tracheae^24^, metaleg trachea^17^
MtSp	metathoracic spiracle	^1,29^/posterior thoracic spiracle^7^
Pe	peritreme	^1,7,8,17,25,26,30,45^
PPb	posterior prothoracic branch	newly introduced
PtLt	prothoracic leg trunk	prothoracic leg tracheae^24^, proleg trachea^17^
SpO	spiracular opening	^7^/spiracular aperture^1^, atrial orifice^3^
SpOa/SpoP	anterior/posterior spiracle opening	spiracular aperture^1,8^
SpOS	spiracular opening spine	used as taxonomic character in some insects e.g. Coccidae^56^
VAC	ventral abdominal commissure	^29^/transverse ventral commissures^1^, ventral commissure of abdomen^24^, abdominal ventral commissure^17^, ventral tracheal commissure^45^.
VCt	ventral cephalic trunk	ventral head trunk^1^, ventral tracheae of the head^24^, ventral cervical trachea^55^, ventral cephalic trachea^7^, ventral head trachea^17,25^
VMCb	ventral median cephalic branch	labial trachea^17^
VMsC	ventral mesothoracic commissure	mesothoracic commissure^24^
VMtC	ventral metathoracic commissure	metathoracic commissure^24^
VPC	ventral prothoracic commissure	ventral tracheal commissure^1^, prothoracic ventral commissure^29^, ventral anastomosis^55^
VPt VMst VMtt	ventral prothoracic trunk ventral mesothoracic trunk ventral metathoracic trunk	ventral plurisegmental tracheal trunk^1^, ventral lateral trunk^29^, lateral longitudinal trunk^55^, ventral thoracic trachea^7^, ventral longitudinal trunk^17^
WAtr	atrial wall	^1,7,27,45^

Videos are included as Supplementary information (Supplementary Videos S1–S4) and show 3D volume-rendered animations with details of: the complex web of tracheal tubes (Supplementary Videos S1–S3); relationships with the internal anatomy (Supplementary Video S1); the tracheal tubes and their connections to the spiracles (Supplementary Video S3); and a detail of the metathoracic spiracle and the connecting tracheal tubes (Supplementary Video S4). The lumina of the tracheae are shown in Figs. 6–9 and in Supplementary Videos S1–S3, with a colour gradation code, in accordance with their actual lumen diameter range (1.99–21.93 µm).

**Figure 10:**
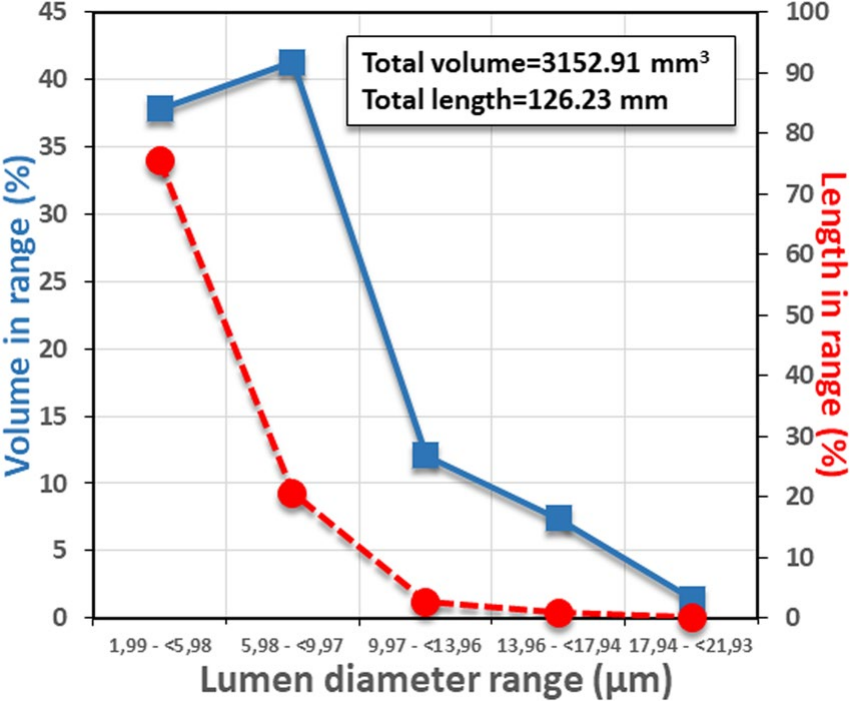
Lumen diameter (µm) distribution ranges of the tracheal tubes and their corresponding percentage of volume capacity. The total volume capacity and total length of the tracheal tubes is indicated.

Lumen size distribution ranges of the tracheal tubes and corresponding percentage volume capacities are shown in Fig. 10. The total volume capacity and total length of tubes are also indicated.

A vxm file with a model of the tracheal system and teguments for use in mobile devices is included as Supplementary 3D model S5.

### Organization of the respiratory spiracle**s**

The tracheal respiratory system opens externally via seven pairs of spiracles (two thoracic [meso- and metathoracic] and five abdominal); these were clearly visible in both light microscopy slide preparations and in micro-CT reconstructed images. The spiracular external openings connect with atrial cavities and tracheal tubes. The mesothoracic spiracles (MsSp) are situated in a latero-ventral position in the pleural membranous zone, anterior to the mesothorax (Figs. 1A,B, 2D, 3A, 5A, 6, 7A, 8, 9A,B,E; Supplementary Videos S1–S3).

The mesothoracic spiracles protrude and each have two elongated spiracular openings: an anterior opening (SpOa) and a posterior opening (SpOp). The tegument extends to form an anterior lip (AlA) and a posterior lip (PlA). Inside each spiracular opening there is a small cavity called the first atrium (Atr1); this is connected to a second atrial cavity (Atr2) through a cribellated plate of tegument, with two longitudinal openings, several pores, and the first filter (Fi1). The second atrium is connected to a third small atrial cavity (Atr3) through another cribellated plate, housing a second filter (Fi2) (Figs. 2A, 3B and 4).

The methathoracic spiracle (MtSp) are located on the pleura in a latero-dorsal position, beneath the elytra (Figs. 1B, 2C, 3A,H, 5A, 6–8 and 9E, Supplementary Video S4). Each methathoracic spiracle (MtSp) has a small spiracular opening connected to the dome-shaped first atrial cavity (Atr1), through a cribellated curved plate, and with a filter (Fi). The first atrial cavity is connected to a second atrial cavity (Atr2) (Fig. 3C–H; Supplementary Video S4).

The abdominal spiracles (ASp) are laterally located on the fore zone of the uniform membranous pleural region (without any epimeral/episternal differentiation) (Figs. 1B,C, 2C, 5A and 6–8; Supplementary Videos S1–S3).

The abdominal spiracles are simpler structures than the thoracic ones, each with a single spiracular opening surrounded by a circular peritreme sclerite (Pe), opening into a barrel-shaped atrial cavity at the bottom of which is a closing apparatus (ClA) that forms a U-shape elastic bar (flexible rod) that constrains the connection with the tracheae (Figs. 1C, 2B and 3I).

### Organization of the tracheal tubes

Three tracheal trunks arise from each mesothoracic spiracle: the latero-ventral prothoracic (LVPt), ventral prothoracic (VPt) and dorsal prothoracic trunks (DPt) (Figs. 3A,B, 4A,E–G, 5–8 and 9A,B,E; Supplementary Videos S1–S3). Each latero-ventral prothoracic trunk is connected through a descendent branch to the prothoracic leg trunk (PtLt) which supplies the prothoracic leg (Figs. 3A, 6–8 and 9A,B,E; Supplementary Videos S1–S3; Supplementary 3D model S5). Descendent branches on both sides are interconnected by a ventral prothoracic commissure (VPC) before supplying the prothoracic legs (Figs. 5, 8B,C and 9D; Supplementary Videos S1–S3; Supplementary 3D model S5).

The ventral prothoracic trunks run into the head, joining to form the ventral cephalic trunk (VCt). A thin branch is derived from each ventral prothoracic trunk; each branch joins to supply the ventral median cephalic branch (VMCb) which runs ventrally into the head (Figs. 3A, 5–8; Supplementary Videos S1–S3; Supplementary 3D model S5).

The dorsal prothoracic trunks are connected by a dorsal cephalic commissure (DCC) (Figs. 8 and 9A–C; Supplementary Video S1; Supplementary 3D model S5). From each side of the dorsal cephalic commissure two thin tracheae emerge, the dorso-median cephalic tracheae (DMCt) (Fig. 9C; Supplementary Video S1; Supplementary 3D model S5), which run parallel and very close to each other in a median-dorsal position, diverging only at the tip (Figs. 3A, 5, 6B, 7, 8 and 9A–C; Supplementary Videos S1 and S2; Supplementary 3D model S5). The dorsal prothoracic trunks run forward into the head via the dorsal cephalic trunks (DCt). The posterior prothoracic branch (PPb) and the anterior prothoracic twisted branch (APb) are derived dorsally from the dorsal prothoracic trunk (Figs. 3A, 6–8 and 9A,B,D; Supplementary Videos S1–S3; Supplementary 3D model S5).

The dorsal (DMst), lateral (LMst) and ventral (VMst) mesothoracic trunks are derived backwards from the dorsal prothoracic trunk, the ventral prothoracic trunk, and anteriorly and close to the mesothoracic spiracles, respectively (Figs. 3A, 5–8 and 9A,B,E; Supplementary Videos S1–S3; Supplementary 3D model S5). The arc-shaped mesothoracic trunk (AMst) is derived upwards and backwards from the ventral mesothoracic trunk, close to the mesothoracic spiracle. The arc-shaped mesothoracic trunk meets the metathoracic spiracles and from its dorsal arc several branches emerge dorsally (Figs. 3A, 5–7, 8A and 9E; Supplementary Videos S1–S3); one of these branches emerge dorsally and backwards to supply the elytra (Fig. 5B,C). A descendent branch supplying the mesothoracic leg trunk is derived from each ventral mesothoracic trunk (MsLt) (Figs. 3A, 6, 7, 8A and 9E; Supplementary Videos S1–S3; Supplementary 3D model S5).

In the metathorax the lateral mesothoracic trunk (LMst) and the latero-ventral metathoracic trunk (LVMtt) (which runs laterally from the spiracle towards the ventral section, connecting with the ventral trunk) converge with the metathoracic spiracle, through a short lateral tube (Figs. 3D,H and 6–8; Supplementary Videos S1–S4). There are three main longitudinal tracheal trunks: the dorsal metathoracic trunk (DMtt), derived from the dorsal mesothoracic trunk (DMst); the lateral metathoracic trunk (LMtt); and the ventral metathoracic trunk (VMtt), derived from the latero-ventral metathoracic trunk (LVMtt). A branch that bifurcates to supply the hindwings is derived dorsally and backwards from the dorsal metathoracic trunks (Fig. 5B,C). A descendent branch that supplies the metathoracic leg trunk (MtLt) is derived from the lateral metathoracic trunk (Figs. 3A,F–G,I, 5B, 6–8 and 9E; Supplementary Videos S1–S3; Supplementary 3D model S5). The dorsal metathoracic trunks are divided into a lateral (DMtt-1) and a medial (DMtt-2) parallelised branches (Figs. 7B and 8B; Supplementary Videos S1–S3, Supplementary 3D model S5).

Ventrally, in the mesothorax, the left and right ventral mesothoracic trunks (VMst) meet medially to form a large ventral mesothoracic commissure connecting both sides (VMsC); a similar connection exists in the metathorax, where the latero-ventral metathoracic trunks (LVMtt) converge medially to form a ventral metathoracic commissure (VMtC). Both commissures are medially interconnected by a ventral longitudinal trachea (Fig. 8B,C; Supplementary Videos S1–S3; Supplementary 3D model S5).

The dorsal metathoracic branches (DMtt-1, DMtt-2) converge posteriorly with the lateral metathoracic trunk (LMtt), the ventral metathoracic trunk (VMtt) and the abdominal latero-ventral trunk (ALVt) before connecting with the first abdominal spiracle (ASp1) (Figs. 6–8; Supplementary Videos S1 and S2; Supplementary 3D model S5).

The abdominal spiracles are connected by three longitudinal tracheal tubes, the latero-abdominal tracheae (LAt). The ones connecting the first (ASp1) and second (ASp2) spiracles are thicker, and interconnected dorso-ventrally with the abdominal latero-ventral trunks (ALVt). Both sides are connected by a double ventral transversal abdominal commissure (VAC) at the level of the fourth (ASp4) and fifth (ASp5) spiracles. Two parallel genital tracheae (Gt) are derived from the fifth abdominal spiracle (Figs. 1B, 3I, 5B and 6–8; Supplementary Videos S1 and S2; Supplementary 3D model S5).

Two parallel tracheae, the long abdominal latero-median tracheae (LALMt), are derived forwards from the right fourth abdominal spiracle; they ascend dorsally at the level of the first abdominal spiracle, bend backwards, and progressively curve to the left to occupy a median position (Figs. 3I, 6B, 7 and 8; Supplementary Videos S1 and S2; Supplementary 3D model S5).

## Discussion

The external openings of the spiracles can represent a risk of water loss and the entry of particulate matter, pathogens, and/or parasites. However, insects have developed hairs, sieve filters and closing mechanisms (such as lips or valves) to minimise these issues^1,2,6,15,42^. Our results show the presence of protective mechanisms in all the spiracles, including filtering sieve plates and the closing apparatus.

Tubular tracheal systems in insects are formed during embryogenesis as a series of segmental invaginations of the integument. Up to three thoracic and nine abdominal pairs of spiracles may exist in embryos, though this number is always reduced prior to hatching; further reductions may occur in endopterygotes during metamorphosis (resulting in a maximum of two thoracic and eight abdominal pairs, in adults). The spiracles of the prothorax disappear during development and those of the mesothorax migrate forward to an antero-lateral position on the prothorax^1,2,4,8,15,17,18^. Thus, even when they are originally ‘mesothoracic’ spiracles, due to the acquired new anatomical location, actually they appear situated in a prothoracic position, as shown in Fig. 5A.

In the beginning of the twentieth century, Fuchs^43^ considered the number of abdominal spiracles as a taxonomical characteristic that varied according to sex. However, in the coffee berry borer we observe that both sexes have the same number of abdominal spiracles.

To assist in ventilation, many insects have air sacs (dilations of the tracheal tubes) that force ventilation when they are compressed by movement of the surrounding muscles^1,4,6,8,10,12–17,24,25,44^. However, some coleopteran species have reduced or no air-sacs at all^9,29,31^. Our study shows that the coffee berry borer has very small air sacs in the abdominal lateral trunks, close to the spiracles; these were particularly visible close to the third and fifth abdominal spiracles, both with light microscopy (Fig. 1Ab) and micro-CT rendered images of the tracheal lumina, where they appear as small dilations close to these abdominal spiracles (Figs. 6–8).

It has also been shown that compression of the pronotal main tracheal trunks (dorsal and ventral prothoracic trunks) forces ventilation and exchange of gases in the tracheal tubular system^3,7^. Cross-sections of these compressible trunks show elliptic-shaped lumina to make them easily collapsible^1,8,18,24^ as seen in Fig. 9D. Hence, due to their proximity, the mesothoracic spiracles must have an important functional role in tracheal ventilation, with large volumes of gases passing through them. In fact, the tracheal tubes arising anteriorly to the mesothoracic spiracles supply the prothoracic and proventricular muscles and the forelegs, and via cephalic derivations they also supply the brain, the subesophageal ganglion, and the mouthparts (Fig. 5B).

Confirming their key functional importance, the mesothoracic spiracles are by far the most complex, with three consecutive filter plates and three atrial cavities. Moreover, the external openings have two mobile lips, one anterior and another posterior, to close the anterior and posterior external spiracular openings, respectively (Fig. 4). Spiracles situated underneath the elytra (mesothoracic and abdominal) are more protected; we did not observe any filters in the abdominal spiracles (Figs. 1C, 2B and 3I). In contrast, the metathoracic spiracles, which are more exposed because the metathorax moves in relation to the abdomen, showed a very small spiracular opening (Fig. 3H) and a filter plate (Fig. 3C–H). Tracheal tubes in the meso- and metathorax surround and supply the anterior midgut, and the middle and hind legs. Extensions from the ventral pro- and mesothoracic commissures, together with anterior prolongations of the meso- and metathoracic commissures, supply the complex ganglion (resulting from the fusion of the thoracic and abdominal ganglions^1^) (Fig. 5B; Supplementary Video S1; Supplementary 3D model S5).

The U-shaped bar of the closing apparatus of the abdominal spiracles, first described by Snodgrass^1^, and observed by many authors^2,4,8,43^, are almost identical to those described for the whirligig beetle *Dineutes indicus* (Coleoptera: Gyrinidae)^7^. Muscles operating the closing apparatus of the abdominal spiracles have been observed previously^2,4,8,16^, as well as the muscles operating the closing apparatus of the mesothoracic spiracle in the coffee berry borer^28^. The latter was not visible in Fig. 2 because the specimen had been treated with KOH, nor were they observed in micro-CT rendered images.

The general organisation of the tracheal system in the coffee berry borer is similar to that described previously for others coleopteran species, such as two tenebrionids, the flour beetle (*Tribolium anaphe*)^29^ and the mealworm beetle (*Tenebrio molitor*)^17^. Thus, the dorsal cephalic commissure is clearly visible in the coffee berry borer (Figs. 7B, 8 and 9A–C) but the ventral cephalic commissure was not visible. Transversal fine tubes, close to each other, were observed in the positions where other species have the ventral commissure, but when observed in detail they did not join to form a continuous commissure (Fig. 9B). Moreover, the long abdominal latero-median trachea (LALMt; also termed the ‘long median trachea’), which is normally located on the left side of the animal^29^, was on the right side in the coffee berry borer. This may be because the authors^29^ drew it from a microscope view and thereafter the position was inverted as a specular image, or the position in that species is different to what we observed in the coffee berry borer. Furthermore, in *T. anaphe* the long abdominal latero-median trachea appears as a single trachea when running anteriorly and is divided into six branches when it runs posteriorly; in the coffee berry borer it appears as two tracheal tubes along its entire length (Figs. 6B, 7 and 8; Supplementary Video S4; Supplementary 3D model S5).

Classically the tracheal system is considered to consist of two dorsal longitudinal trunks, two lateral trunks, and two ventral trunks, connected by transversal tubes^2,4,6,8,12,15,17,18^. In the coffee berry borer, it is, more or less, similar to this in the head and thorax. On each side of the abdomen there are three longitudinal latero-abdominal tracheae (LAt) that at first sight, could be interpreted as the dorsal, lateral, and ventral trunks described classically. In fact, previous studies have shown that the dorsal trunks should run along the dorsal vessel (heart) but, in the coffee berry borer, the dorsal latero-abdominal tracheae are situated far away from the dorsal vessel. We observed that the long abdominal latero-median tracheae (LALMt) followed the posterior-midgut and gonads when running forward, while passing over the hindgut (rectum) just below the dorsal vessel when running backwards (Fig. 5B)^41^. Therefore, the long abdominal latero-median tracheae play an important role in supplying the digestive tract, gonads and heart in coffee berry borer. This would explain the presence of small air sacs close to the spiracles for increasing abdominal ventilation. On both sides, the abdominal tracheal system runs posteriorly as two parallel tracheal tubes (genital tracheae); in females these extend to the reproductive system, supplying the ovipositor musculature.

Although many of the anatomical details have been observed and/or described and named in other insect species, we are the first to describe and name new anatomical details. Of note, despite a recent study renaming the ventral abdominal commissures as abdominal ventral commissures^17^, this terms has been used previously by other authors in different insect species^1,24,29,45^ (Table 1).

As identified by Iwan *et al*.^36^, using the same microtomography instrument as us, the small voxel size achieved permits visualization of tracheoles. However, as tracheoles are filled with tracheolar fluid, through which interchange of gases occurs^2,4,8,46^, the methodology is unable to reconstruct the tubes in the same way as gas-filled tube cavities can be reconstructed. In fact, very fine tracheal tubes (up to 1.99 µm diam.) were reconstructed in the coffee berry borer, but nothing narrower could be visualized. Wigglesworth^46^ experienced the same problem when studying the tracheal system of a dragonfly (*Aeschna*) under light microscopy. He observed that when the tracheoles were filled with fluid they were not visible, but by eliminating the tracheolar fluid using a hypertonic solution of potassium lactate, the problem could be solved. Unfortunately, potassium lactate would not solve the problem because it would create other non-tracheolar empty spaces introducing noise into reconstructions.

In recent micro-CT studies of the mealworm beetle tracheal system^9,36^ the minimum diameter of tracheal tubes that could be reconstructed was 25 µm, which is significantly greater than the smallest lumina we reconstructed (1.99 µm), and also greater that the largest lumina we reconstructed (21.92 µm) in the coffee berry borer, which is ten times smaller. However, the largest tracheal tubes of the mealworm beetle have approximately 20 times larger diameters (400 µm) than those of the coffee berry borer, the largest of which are the dorsal and ventral prothoracic trunks that are collapsible to facilitate ventilation (Fig. 8D). Avoiding large diameter tubes could be an adaptation in such a small insect as the coffee berry borer. In fact, to our knowledge, no collapsible tracheae have been reported in mealworm beetles in any studies of its tracheal system^9,31^.

Most of the tracheae we observed have very narrow lumina: the narrowest tubes (lumen diameter range:1.99–6 µm) accounted for more than 75.5% of the total length of tubes and 37.8% of the total volume; all tubes with lumina ≤9.97 µm accounted for 96.6% of the total length and 79.1% of total tracheal volume capacity. A similar relationship between tracheal diameter and its occurrence as a proportion of the entire tracheal system has been reported for the mealworm beetle, although the frequency of occurrence decreases as the diameter increased, and the diameter only decreases from 200 to 400 µm^17^. However, we observed a much steeper slope for this relationship (Fig. 10).

The total estimated length of the tracheal tubes was 122.23 mm. As the studied female was ca.1.8 mm in length, this means that the tracheal tubes were 70 times the total length of the body. If we transpose this to a human scale, then a 175 cm long insect (the average height for American males^47^) would have 123 m of tracheal tubes (longer than a football or soccer field).

In conclusion, we have confirmed that the use of micro-CT techniques to reconstruct and study the tracheal tubular system of insects is an extraordinarily useful and reliable technique; the only limitation was the inability to reconstruct the smallest tubes (<1.99 µm). Even though the technique is time consuming (during scanning but especially during reconstruction and visualisation), once the software parameter for a particular species have been determined and verified, it is possible to create task list procedures that may speed up the process^36^.

This is the first complete study on the tracheal system of the coffee berry borer, the smallest insect to be studied so far using micro-CT. Obtaining similar results using classical dissection methods would have been very difficult, if not impossible. Furthermore, micro-CT allowed to reconstruct the actual position and shape of the anatomical structures without any displacement and/or deformation due to manipulation. It has permitted us to measure, quantify and visualise structures from any perspective. Moreover, videos and a 3D model can be displayed on mobile devices which be used in future research, as well as for teaching insect anatomy to students and the public in general.

## Methods

### Insects

For a previous paper in which we first observed the coffee berry borer inside the coffee berry^40^, J.A.T. collected coffee berries (*Coffea canephora* Pierre ex. A. Froehner; Rubiaceae) at a coffee plantation in Vietnam (*Me Linh Coffee Garden*; 11°53′57.39″N, 108°20′51.16″E; 1043 m.a.s.l.). From this collection, an adult female emerged and was used for the micro-CT study.

### Micro-CT scans

The insect was killed by keeping it for 30 min inside a closed plastic container with a piece of cotton impregnated with a few drops of ethyl acetate. It was then glued, using cyanoacrylate, to the tip of a nylon fishing line 200 μm in diameter, as previously described^48^, and immediately scanned using a Bruker SkyScan 1172 microtomograph (Bruker-micro CT, Kontich, Belgium) with a Hamamatsu L702 X-ray source and a Ximea 11 megapixels camera. The setting parameters were as follows: voltage = 45 kV; current = 45 µA; isotropic voxel size = 1 µm; image rotation step = 0.3°; 360° of rotation scan with no filter. This resulted in a scan duration of 2 h:11 min:39 s, and 1202 X-ray images. The specimen was air-dried, stored and 14 months later it was scanned again to compare the dry preserved internal anatomical structures of the tracheal system (Supplementary Video S4) with the original images from ‘fresh’ material. For this latter scan the setting parameters were the same as the first scan except that it was performed with an 180° rotation scan, resulting in a scan duration of 1 h:10 min:33 s, and 642 X-ray images.

### Image reconstruction

The most recent versions of the Bruker Micro-CT’s Skyscan software (NRecon, DataViewer, CTAnalyser) were used for primary reconstructions and the ‘cleaning’ process to obtain datasets on ‘slices’ through the insect as described previously^48^. Amira’s software, v. 6.7.0 (Thermo Fisher Scientific, Waltham, MA)^49,50^ (with the built-in “volrenRed.col” colour filter) was used to obtain volume-rendered images (Figs. 3–5A and 9D; Supplementary Video S4).

To achieve a clean image dataset for the tracheal tubes we modified an existing methodology^36^. Briefly, it consisted of scanning the insect immediately after killing it. In insects, the haemolymph fills the internal cavities and it has a similar transparency to the internal structures when viewed by X-ray. Thereafter, whereas the reconstructed image slices hardly showed the internal structures, the empty spaces that were full of gas (such as the interior of the tracheal tubes) were clearly visible. Thus, the process allowed us to reconstruct these spaces by inverting images from negative to positive and cleaning away the superfluous non-tracheal spaces, to obtain images that contained only the lumen of the tracheae (as described step-by-step in the Supplementary Methods). During the process, the internal spaces in the elytra and hindwing venations were eliminated to facilitate visualisation.

Colour-coded images were created using the 3D analysis plug-in in the CTAnalyser’s customised processing tab and saved to determine structure thickness (or separation) as done previously^51^ and as described in a Bruker microCT method note^52^. We used CTvox (Bruker’ micro-CT’s Skyscan software) to obtain images for Fig. 5B,C and the final rendered images, videos and 3D model for mobile devices, with colour code representations of the lumen diameter of the tracheae (Figs. 6–9; Supplementary Videos S1–S3; Supplementary 3D model S5).

Reconstructed images of the two scans (earlier for the tracheal tubular system, and months later for the internal structures) were co-registered with the software DataViewer following the procedure described in a Bruker microCT method note^53^.

By running the 3D analysis plug-in (in the customised processing tab) of the CTAnalyser software, we could calculate the total volume of the tracheal tubes, as well the ranges according to the lumen diameter (or thickness). Using extrapolation we considered the tracheal tubes as cylinders and from the total calculated volume of each range and thickness it was possible to estimate their length (length = volume/(π(1/2 thicknes)^2^); adding the calculated lengths for each range we could estimate the total length of all the tracheal tubes in the system (Fig. 10).

### Light microscopy study

To study the position and structure of spiracles under light microscopy, a female adult was progressively cleared by submerging it in a 10% KOH water dilution at room temperature. We examined and photographed the coffee berry borer after immersion for 24 h (Fig. 1A) and 48 h (Fig. 1B). Immediately after that, the insect was dissected, and the dorsal (Fig. 2C) and ventral (Fig. 2D) parts of the thorax and abdomen were dissected and mounted on a slide in modified Hoyer’s liquid media^54^. The position and structure of the spiracles in Figs. 1 and 2 were obtained using a Samsung Note 8 smartphone connected to the ocular of a Motic SMZ-168 stereo zoom microscope (Fig. 1A [except a], 1B) and to an Olympus CH-2 binocular microscope (Figs. 1Aa,C and 2).

## Supplementary information

Supplementary information

Supplementary video S1

Supplementary video S2

Supplementary video S3

Supplementary video S4

Supplementary 3D model

## Data Availability

The datasets generated and analyzed during the course of the study are available from J.A.T. upon reasonable request.
