# Supplementary material for: Revealing the respiratory system of the coffee berry borer (Hypothenemus hampei; Coleoptera: Curculionidae: Scolytinae) using micro-computed tomography
Source: Sci Rep. 2019 Nov 28;9:17753. doi: 10.1038/s41598-019-54157-3 (PMC6882887; doi:10.1038/s41598-019-54157-3)
Supplement: Supplementary file 1 — Supplementary information [file 41598_2019_54157_MOESM1_ESM.docx]

**Supplementary Information**

**Revealing the respiratory system of the coffee berry borer (*Hypothenemus hampei*; Coleoptera: Curculionidae: Scolytinae) using micro-computed tomography**

**Javier Alba-Tercedor^1,*^, Ignacio Alba-Alejandre^1^, and Fernando E. Vega^2,*^**

^1^Department of Zoology, Faculty of Sciences, University of Granada, Campus de Fuentenueva, 18071-Granada, Spain

^2^Sustainable Perennial Crops Laboratory, United States Department of Agriculture, Agricultural Research Service, Beltsville, MD, 20705, USA

^*^ Correspondence and requests for materials should be addressed to J.A.T. (email: [jalba@ugr.es](mailto:jalba@ugr.es)) or F.E.V. (email: [Fernando.Vega@ars.usda.gov](mailto:Fernando.Vega@ars.usda.gov))

**Supplementary Methods**

**Procedure to isolate the tracheal system**. A step-by-step explanation of the procedure followed to reconstruct and isolate the lumina of the tracheal tubes.

**1.- LOAD RAW IMAGES INTO THE SOFTWARE CTAnalyser:**


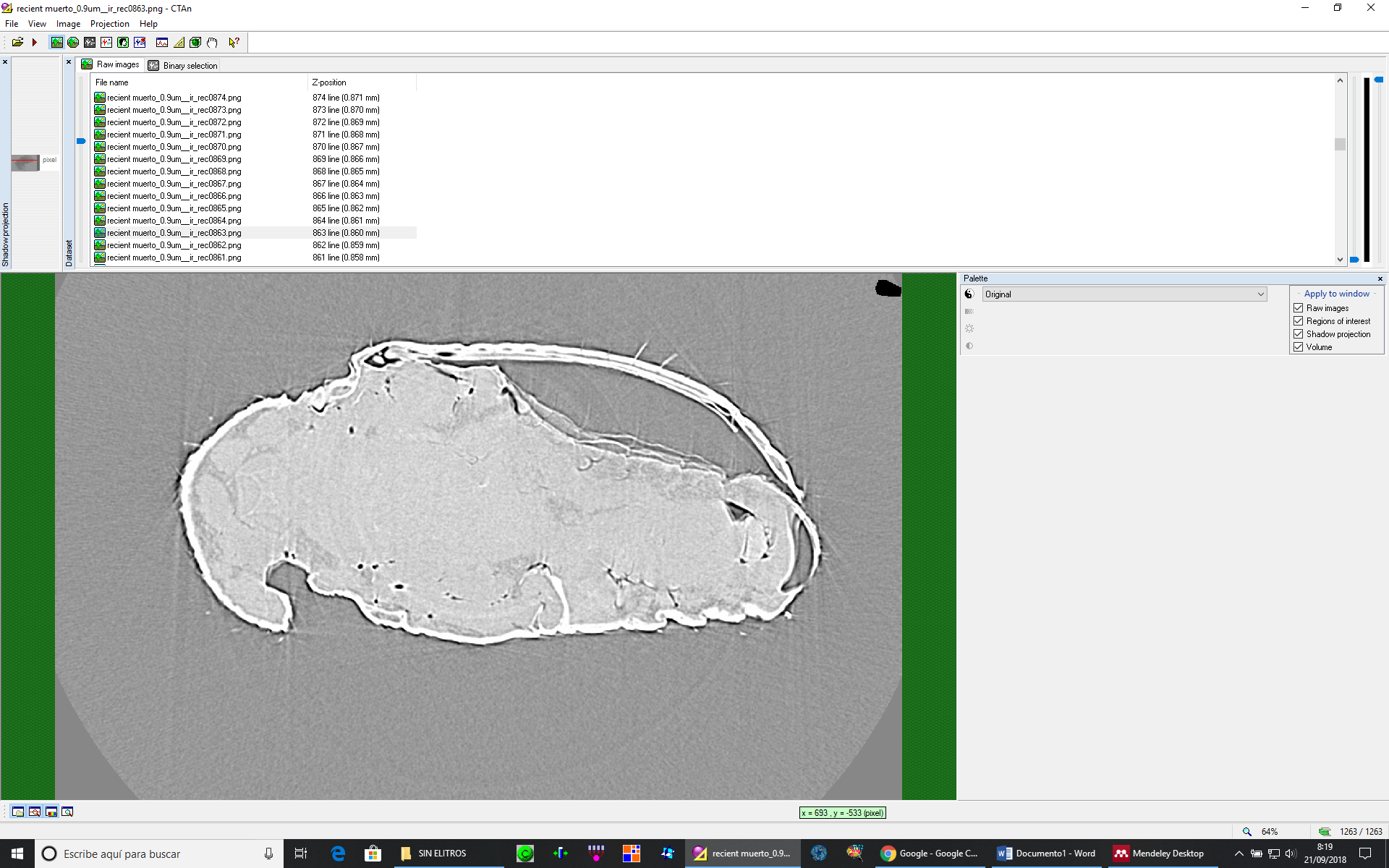


**2.- SEGMENT IMAGES (Threshold):**


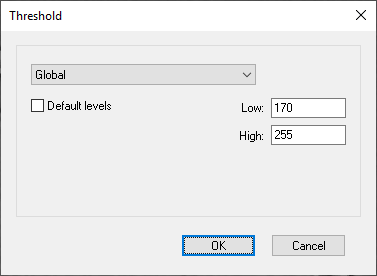

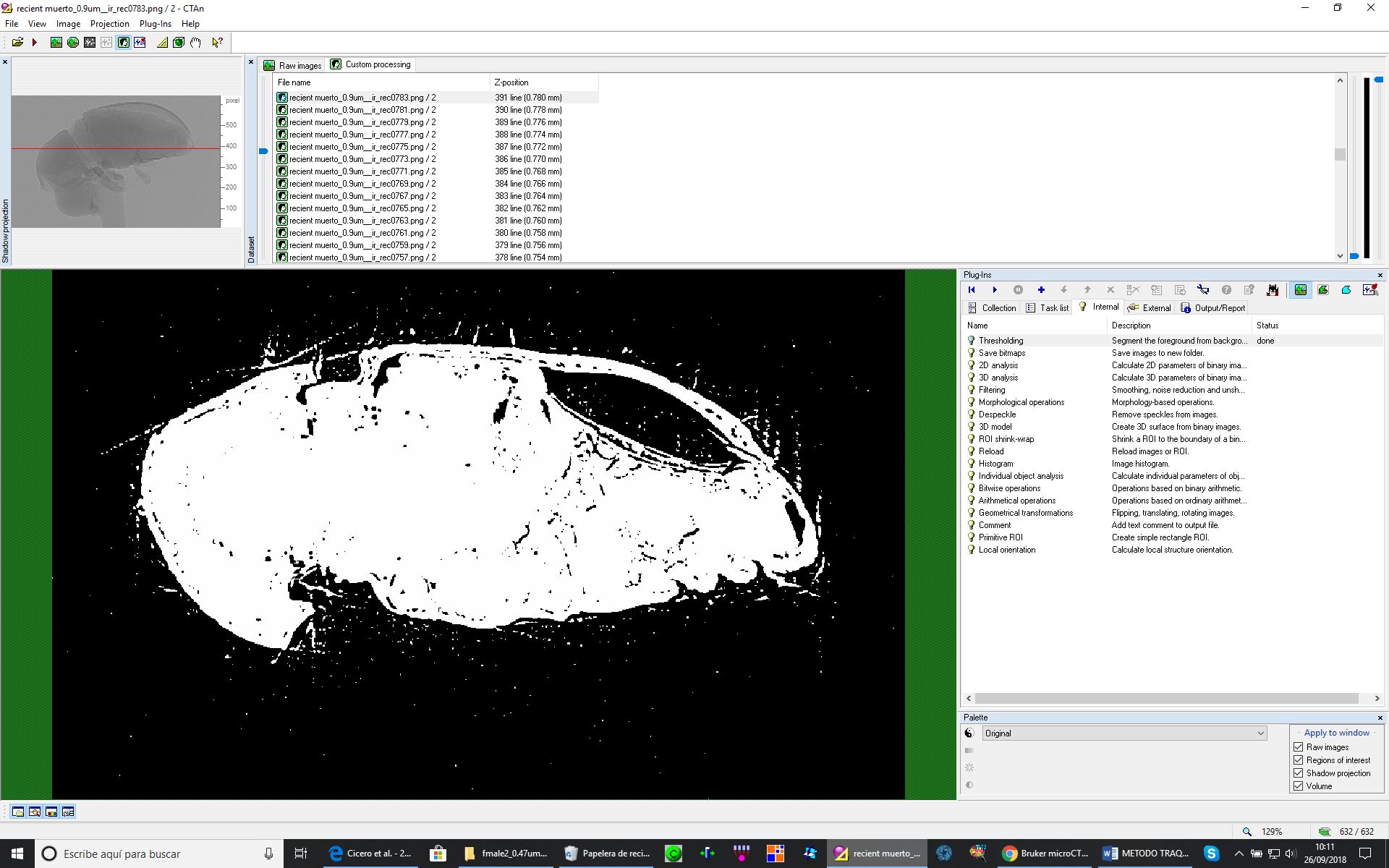


**3.- ISOLATE CAVITIES WITH AIR (mostly tracheae):**

3.1.- Bitwise operations:


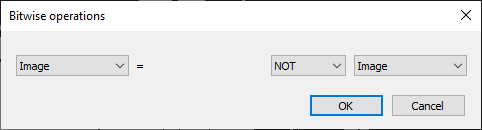

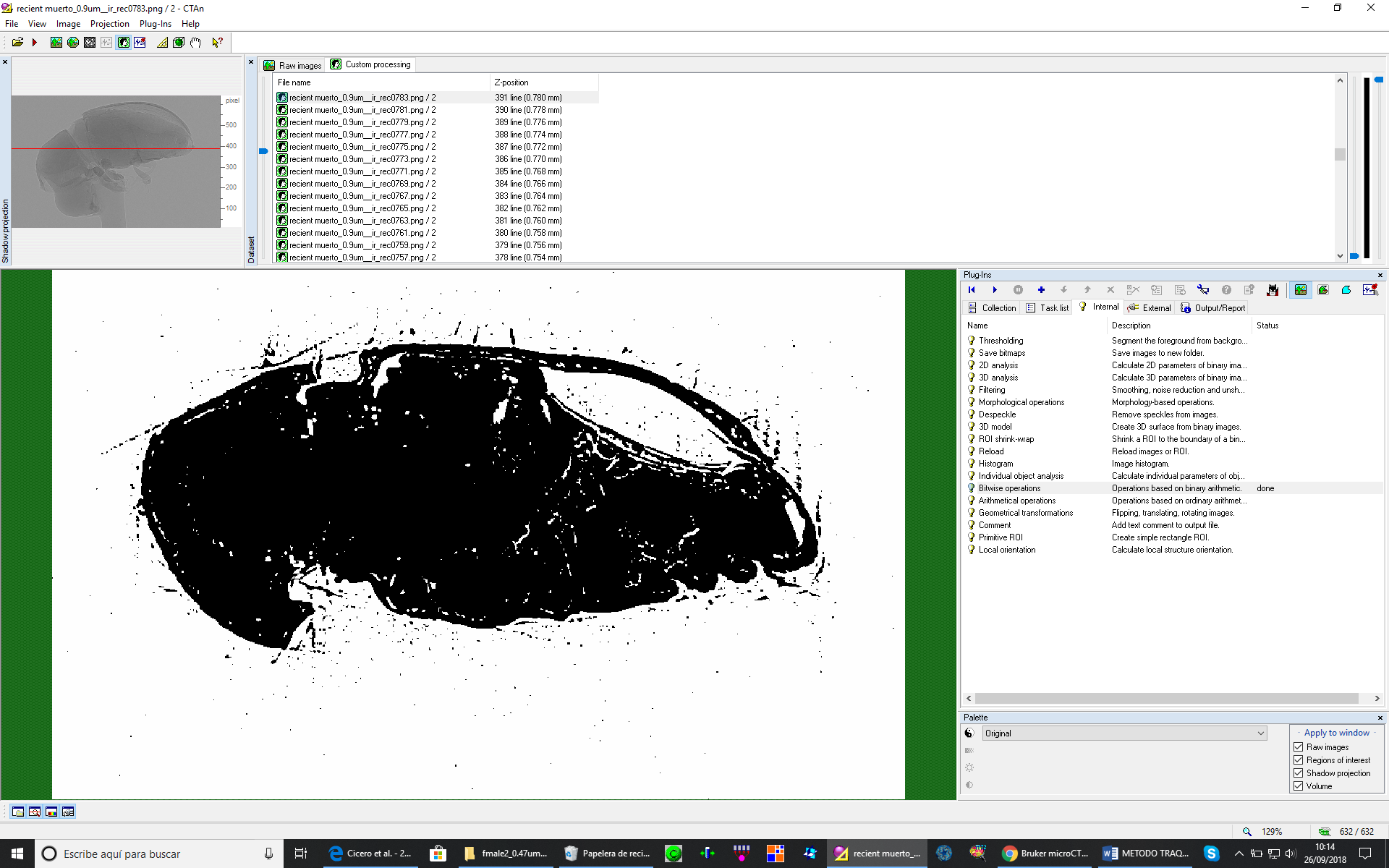


3.2.- Despeckle (Remove outer objects 2D):


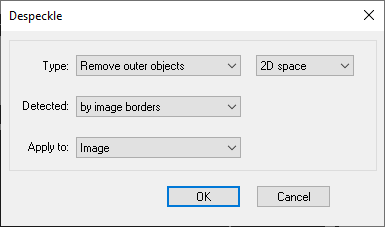

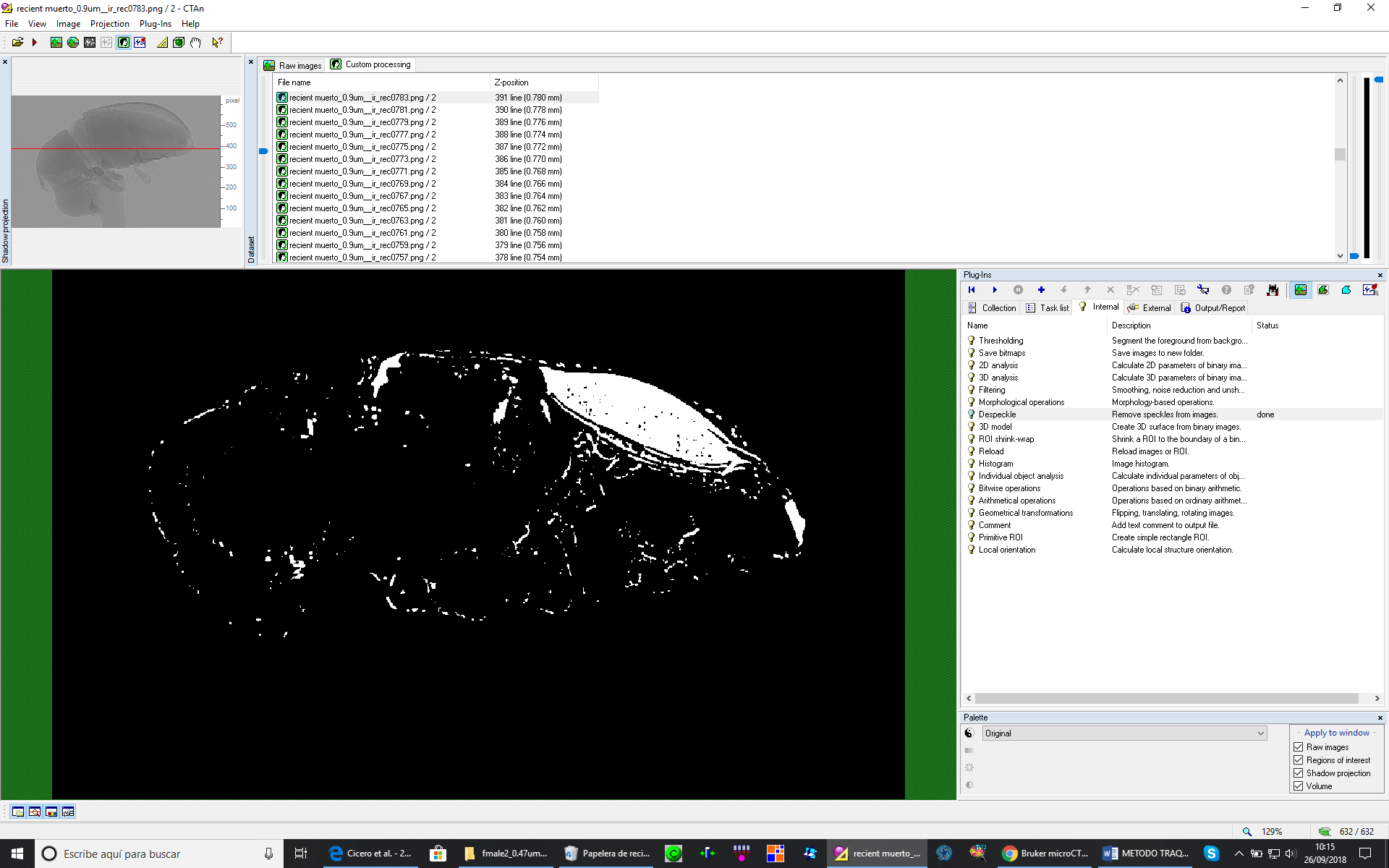


Note that non-tracheal cavities surrounding the external wall still show up and that some big spaces filled with air (e.g., those under the elytra) appear in some cuts:


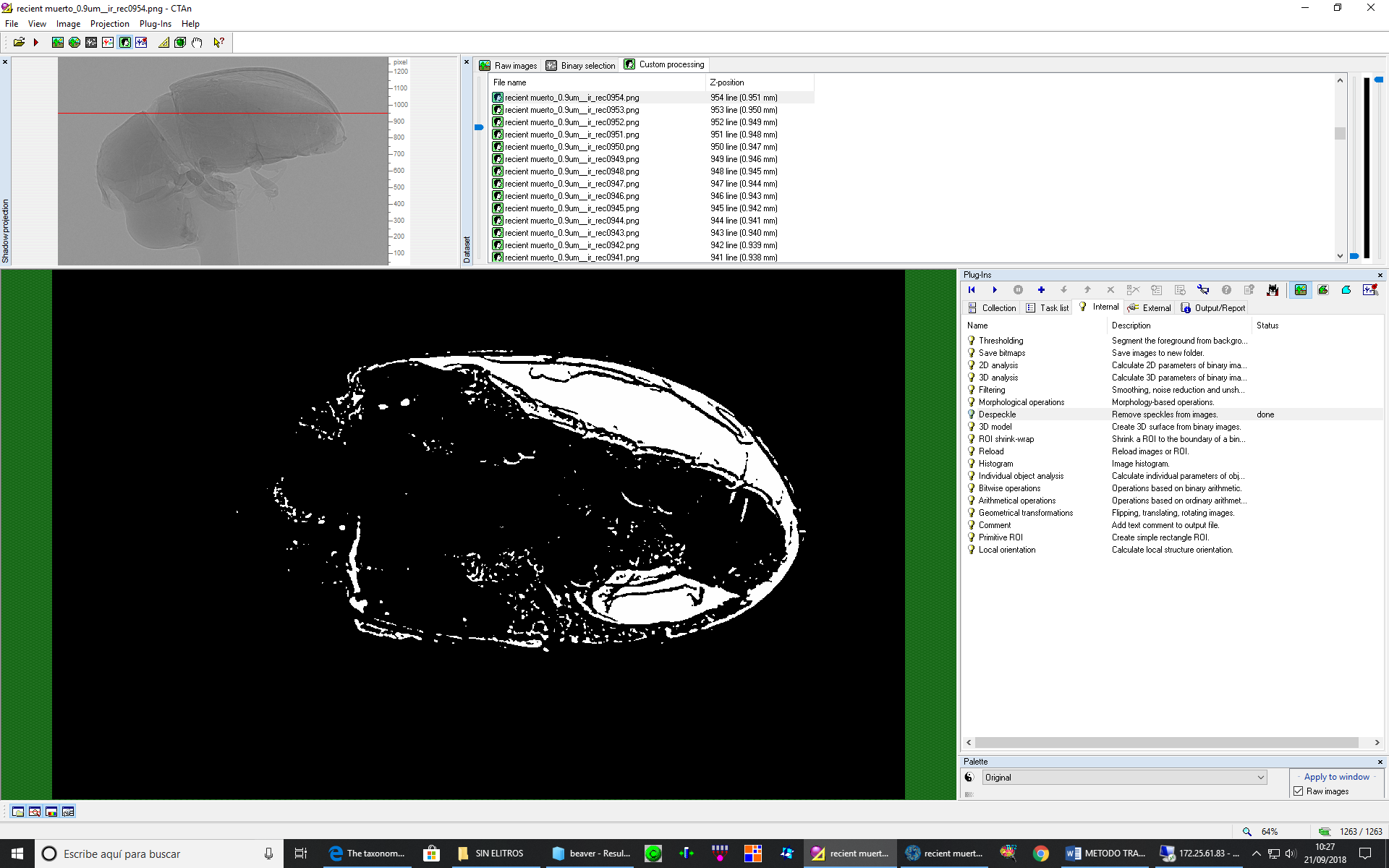

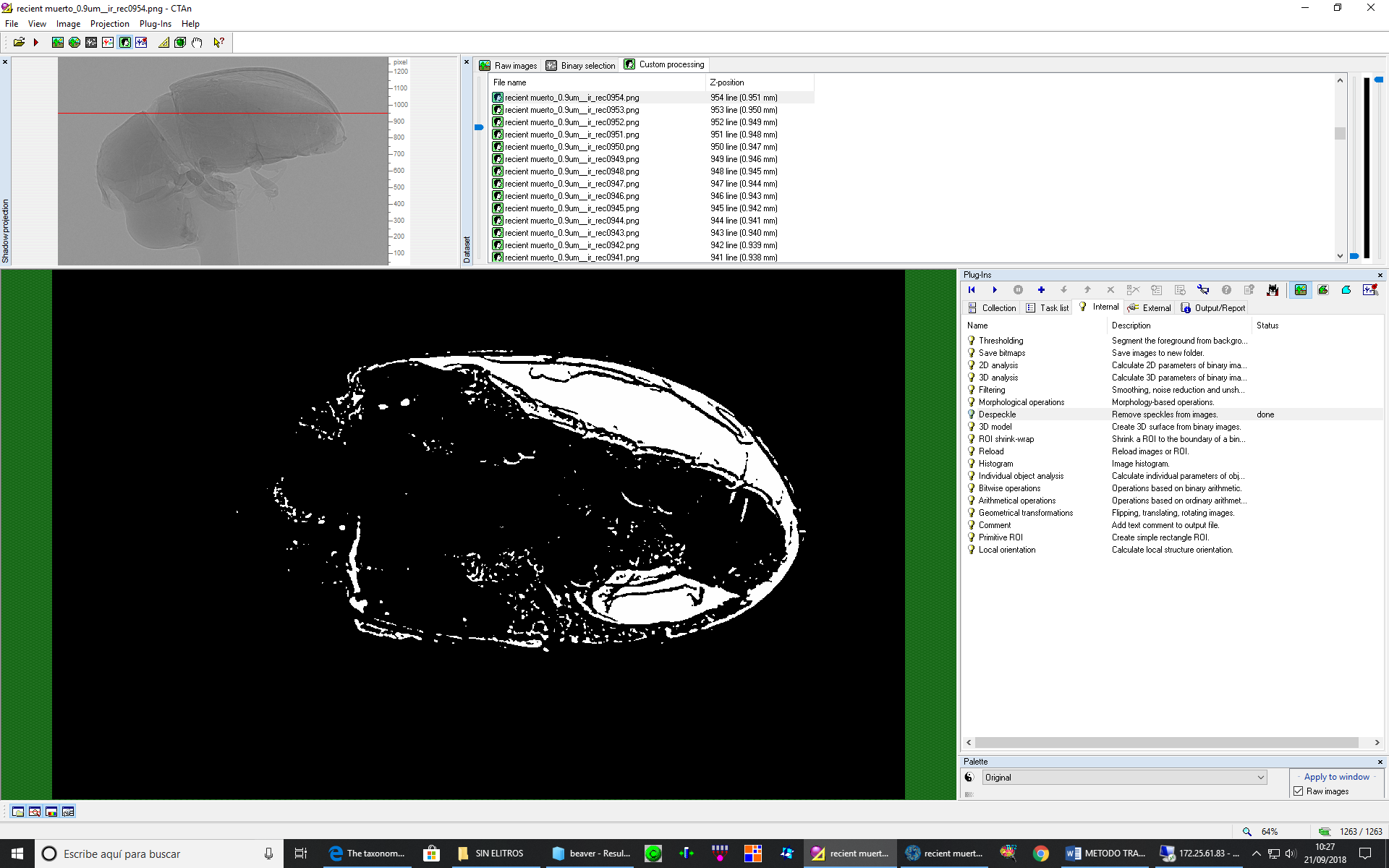


At this point, the volume rendering will show too much non-tracheal space with empty cavities when reconstructed:


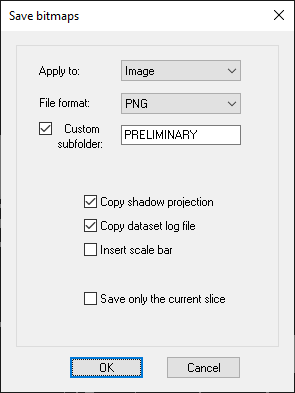

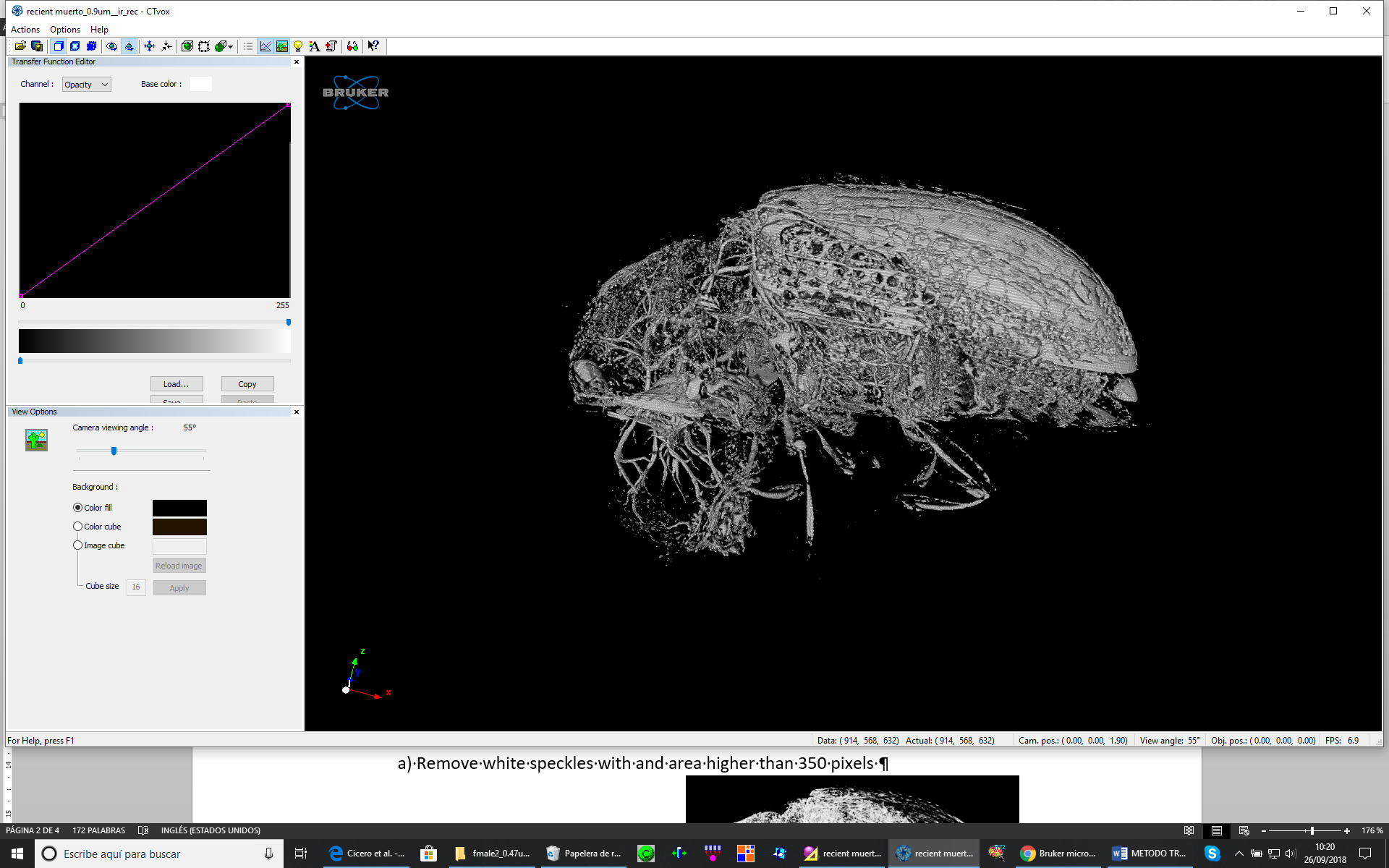


3.3.- Remove the non-desired reconstructed cavities:

3.3.1.-Remove pores from the images (Remove pores 2D):


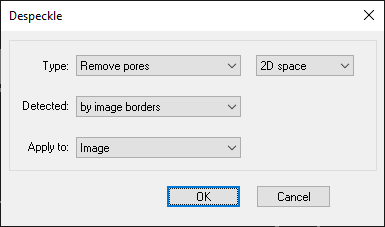

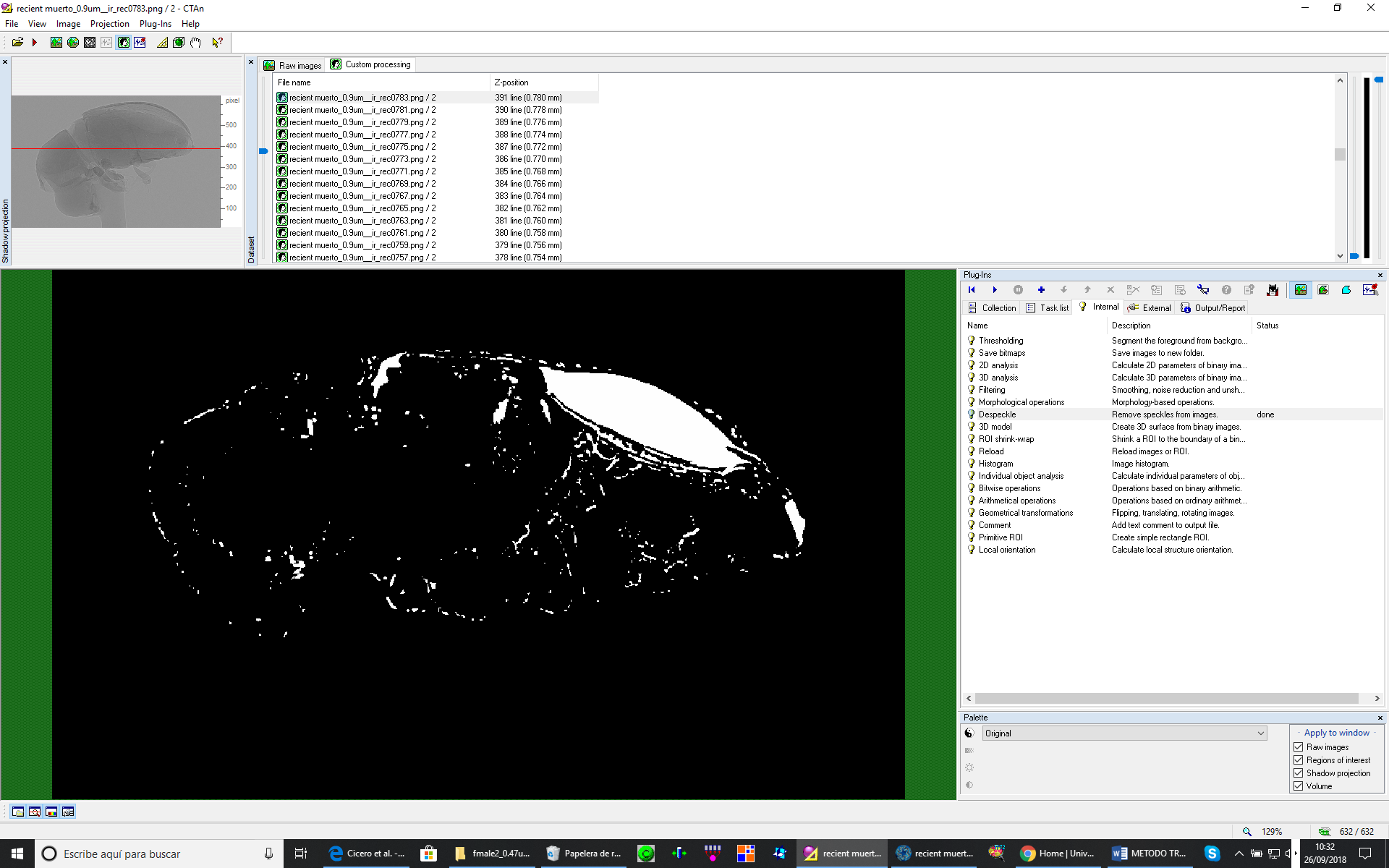


3.3.2.- Remove big white speckles (Despeckle 2D):

After trying different values, we decided to remove white speckles higher than 412 pixels.


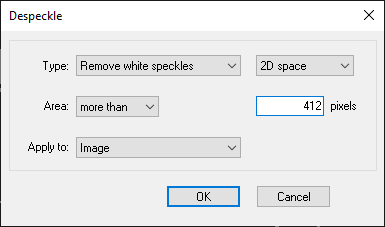

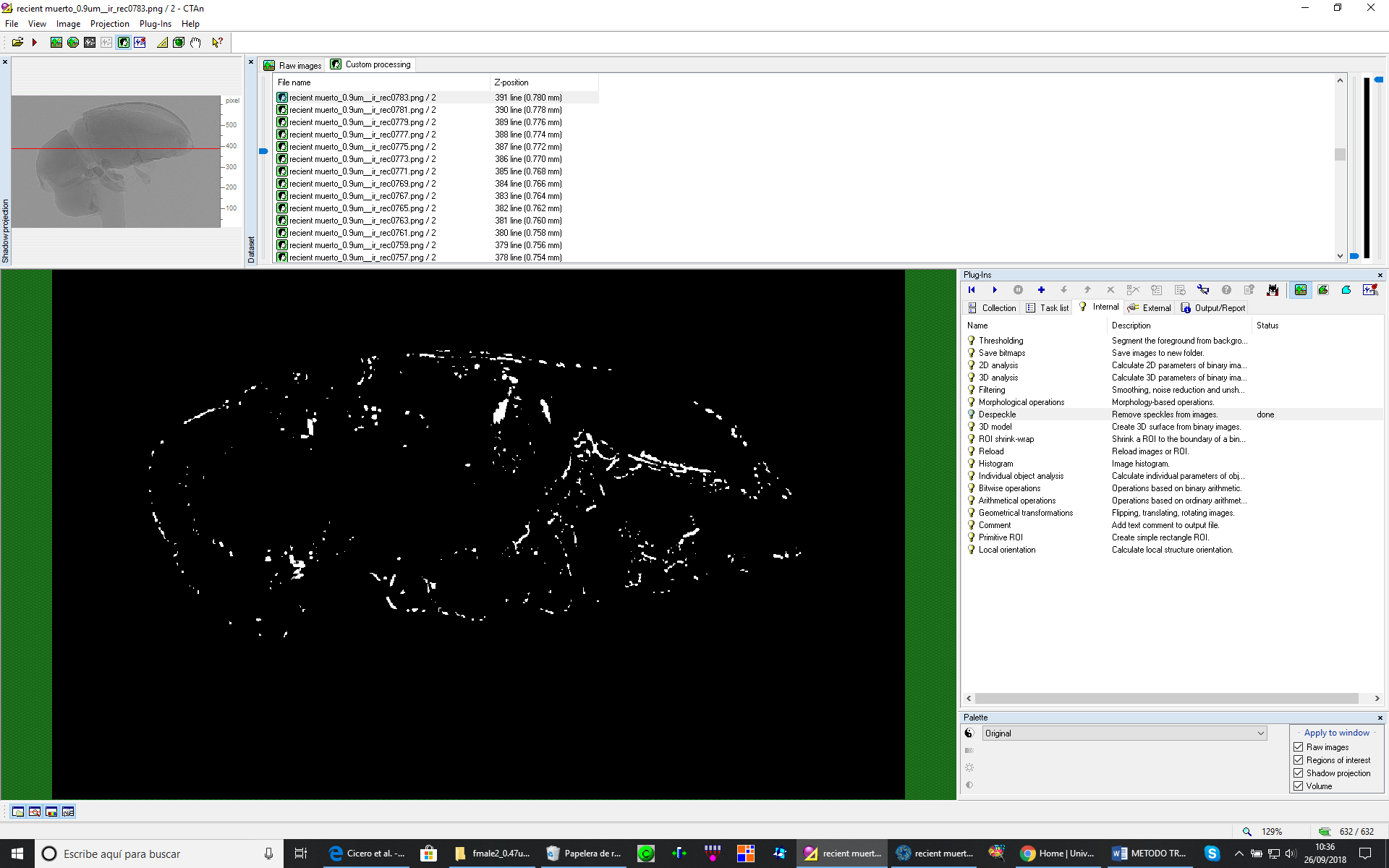


3.3.3.- Eliminate isolated “islands” (Despeckle 3D):

Non-tracheal isolated structures, appear, mostly “outlining” the insect. These can be eliminated by removing white speckles in 3D (after trying different values, the one that worked properly was a value lower than 125 voxels).


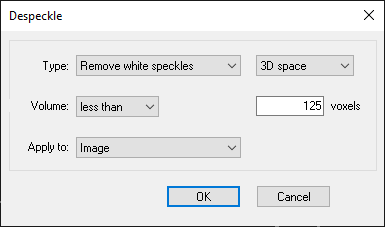

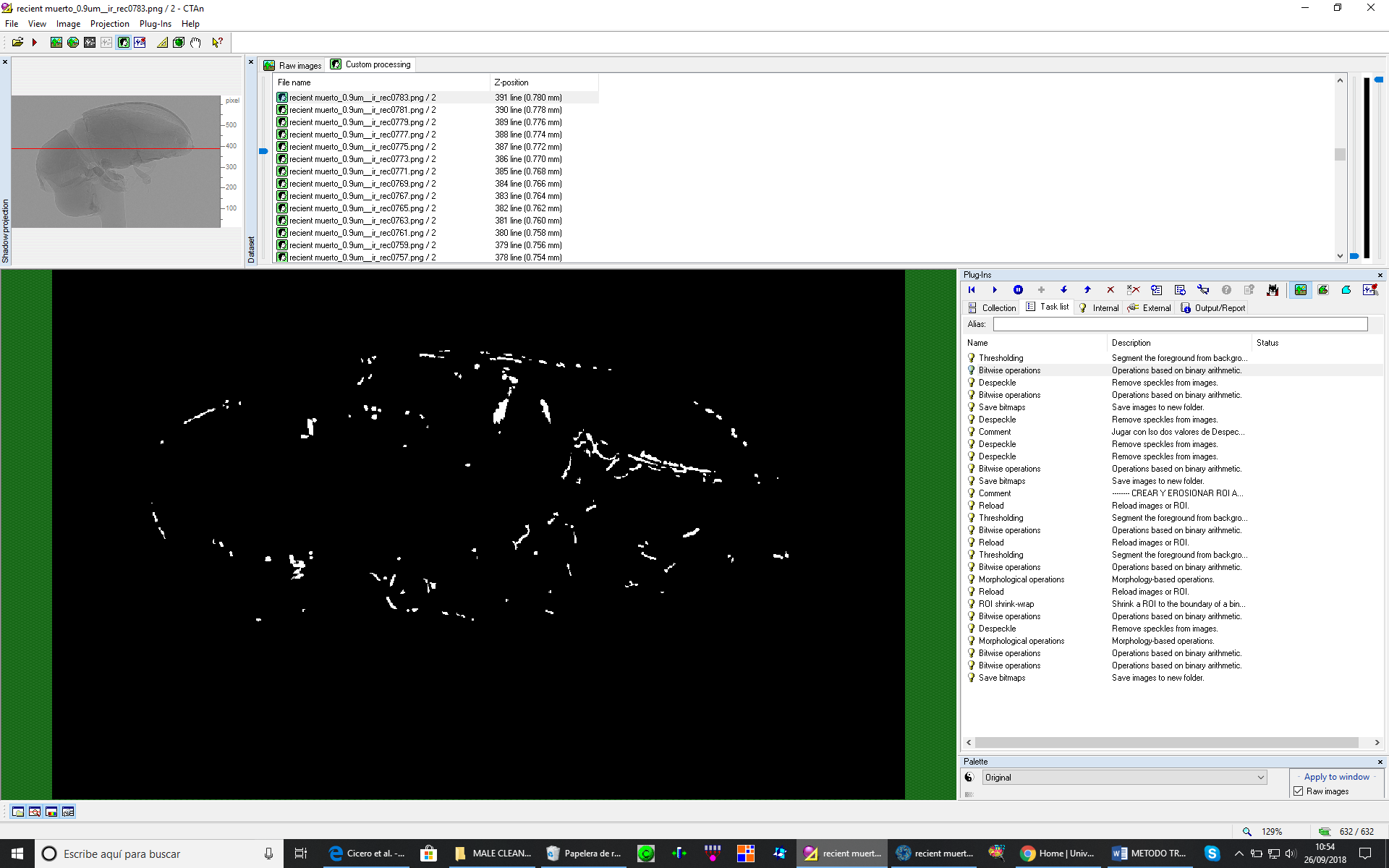


At this point, a cleaner rendered image is obtained but still not clean enough:


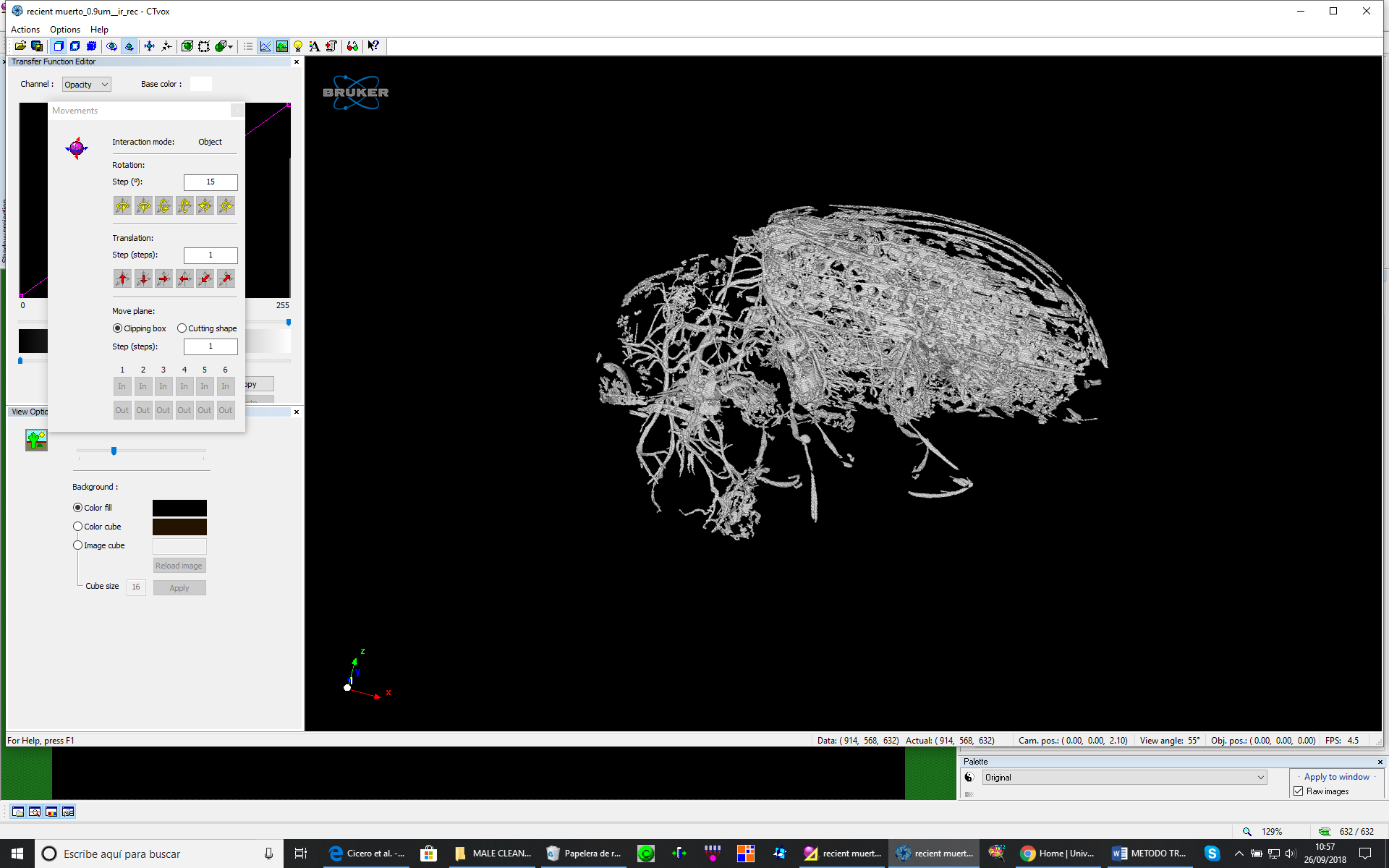


3.3.4.- The resulting images are temporarily saved into the clipboard (Bitwise operations):


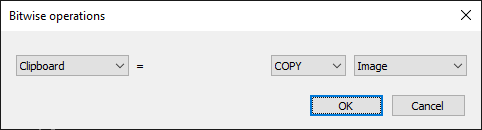


**4.- “CLEAN” THE IMAGES:**

The procedure includes creation of a region of interest (ROI) of the denser tegument covers, which needs to be subtracted from the images stored in the clipboard:

4.1- Reload image (Reload):


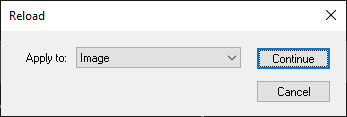


4.2.- Run a thresholding operation (Threshold):


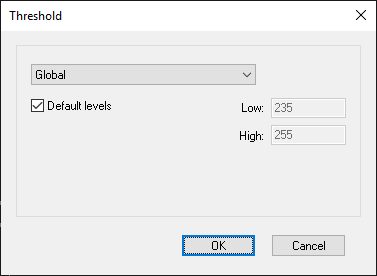


Thereafter, only denser parts (external teguments) are segmented.


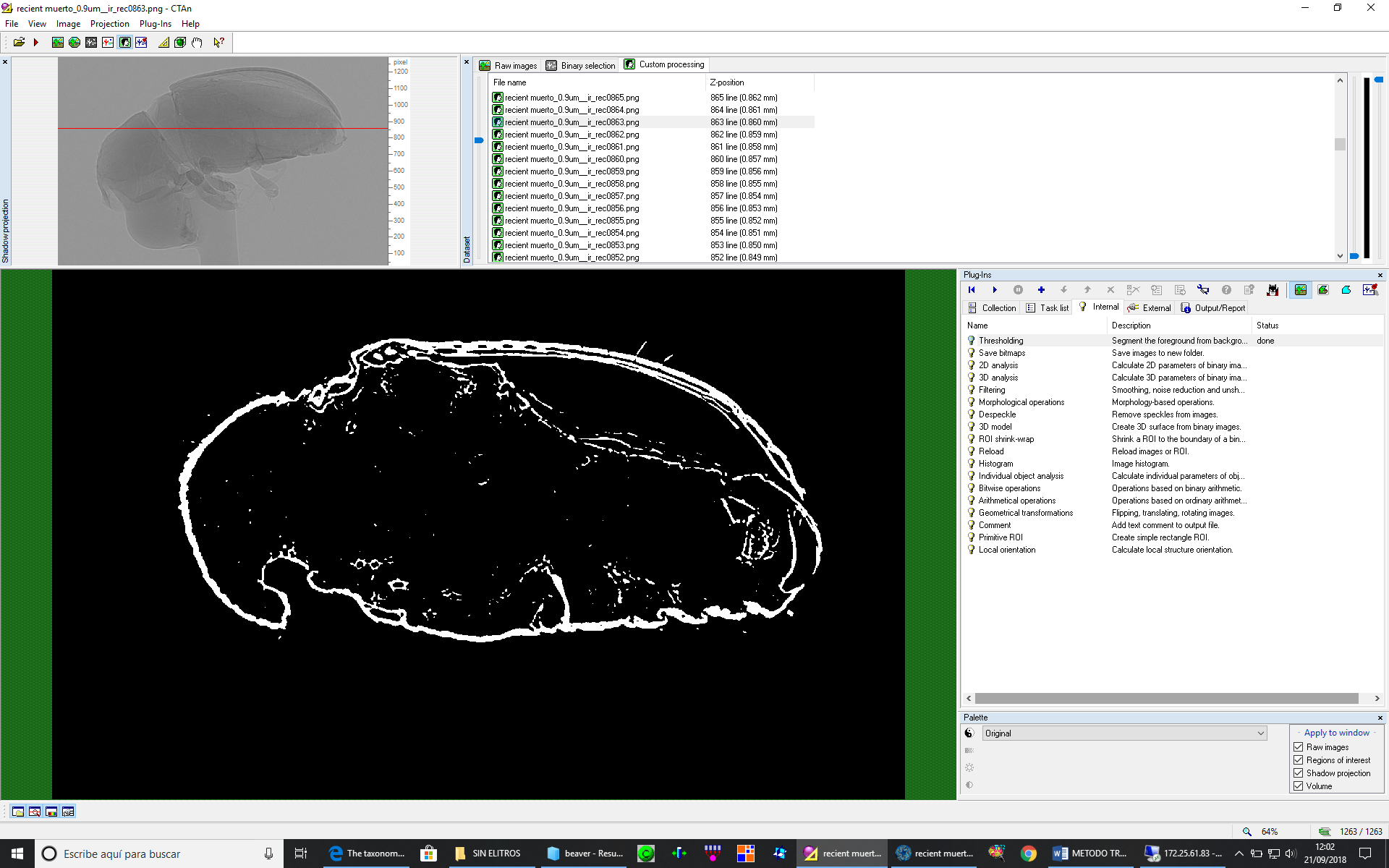


4.3.-To finally get the reconstructed cavities without the external walls, it is necessary to subtract these images to the resulting “clipboard” saved images:

4.3.1.- Copy the segmented image to ROI (Bitwise operations):


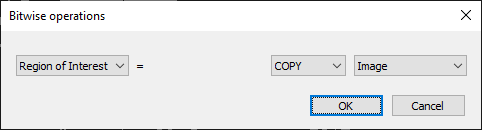


4.3.2.- Reload the image (Reload):


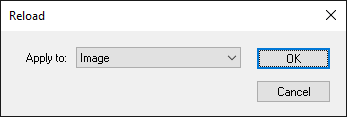


4.3.3.- Segment (Threshold):


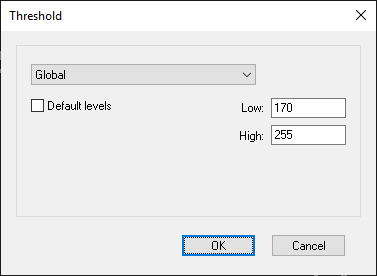


4.3.4.- Subtract the ROI to the image (Bitwise operations):


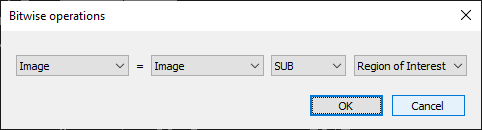


The resulting images have an outlining “halo”:


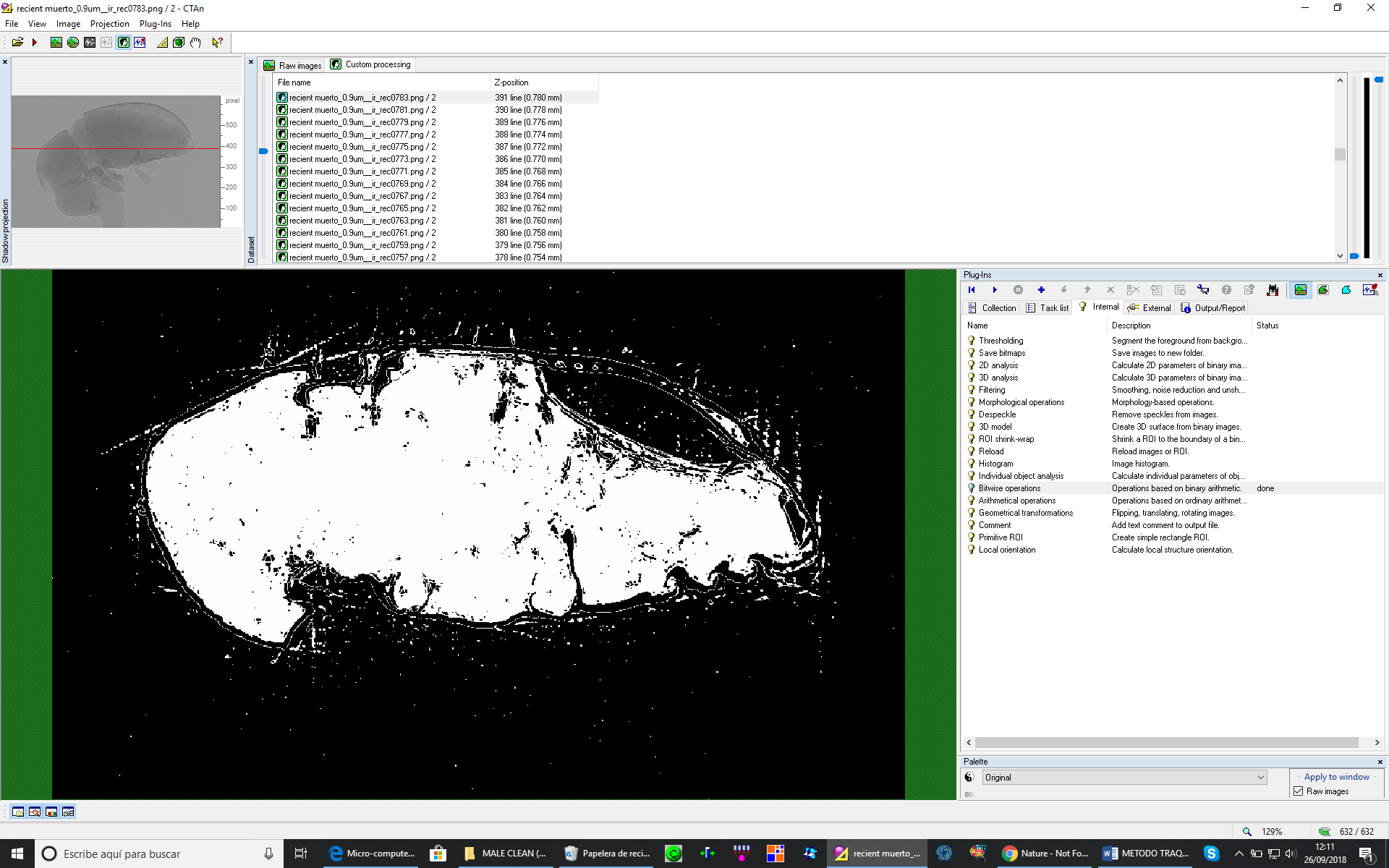


4.3.5.- Eliminate the “halo” (Morphological operations 3D):

To eliminate the “halo” formed around the high density materials (teguments) it is necessary to run an “opening” morphological operation (as reported in Bruker micro-Ct Academy, vol. 2, issue 8, August 2015, <https://www.bruker.com/fileadmin/user_upload/8-PDF-Docs/PreclinicalImaging/microCT/ACADEMY/Bruker_microCT_Academy_2015_issue_8.pdf>)


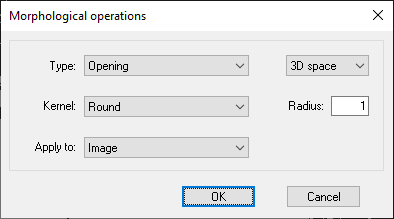

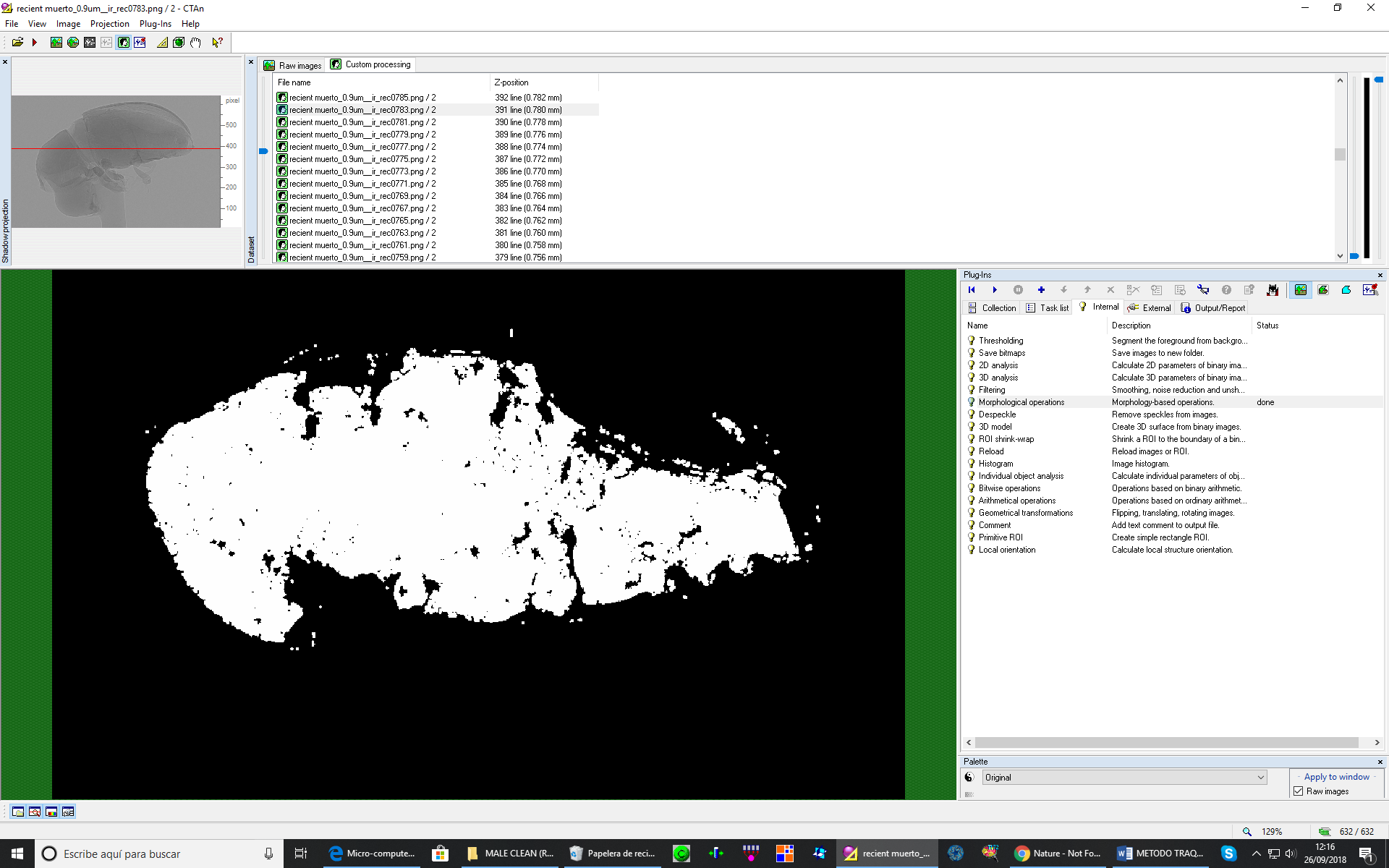


4.3.6.-Reset the existing ROI (Reload):


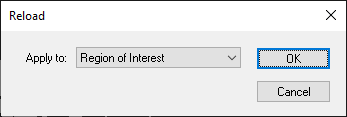


4.3.7.- Create a new ROI around the new image (2D ROI shrink-wrap):


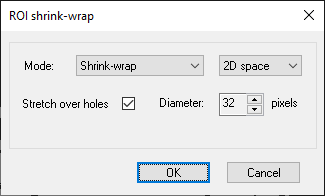

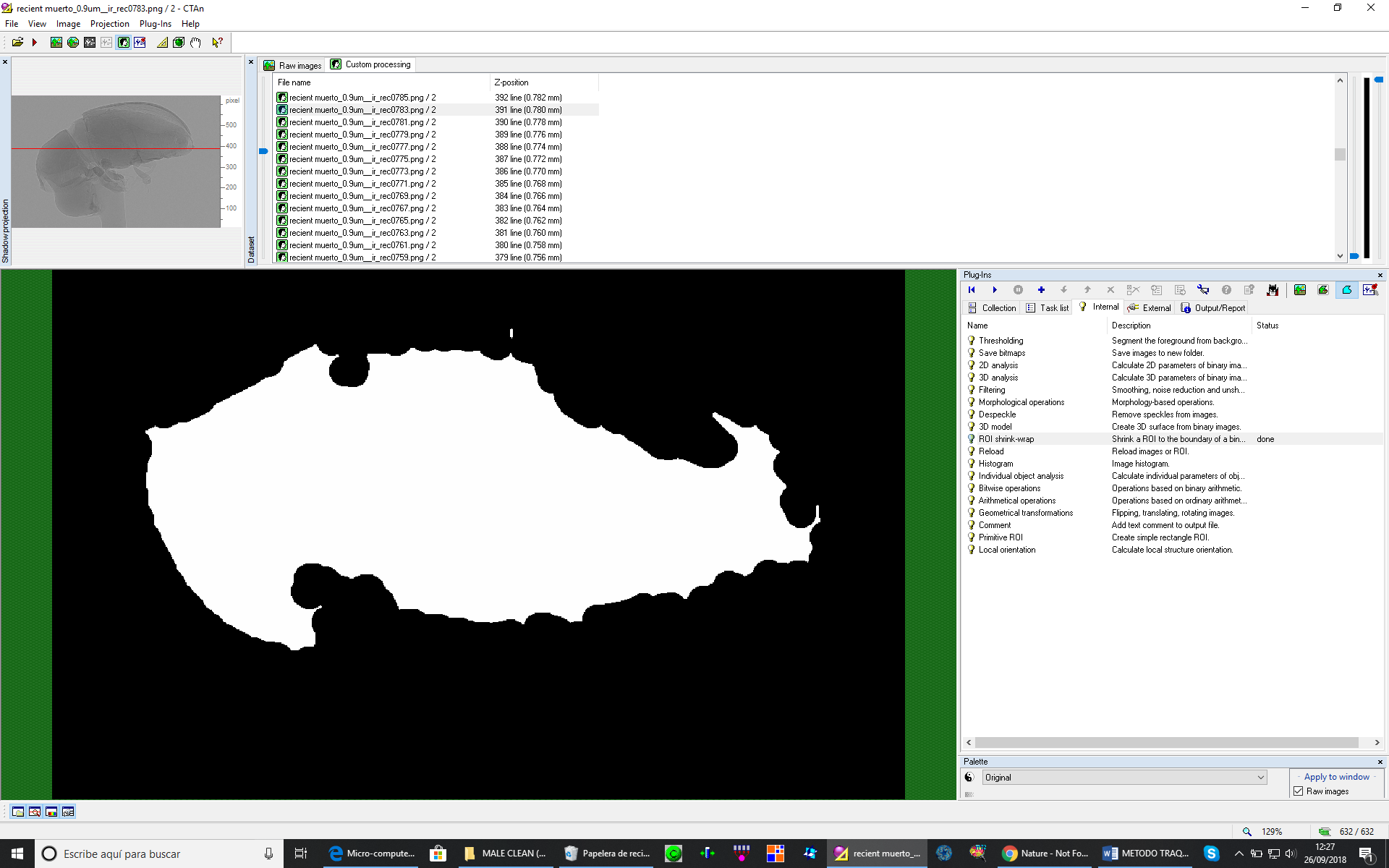


4.3.8.- Copy the ROI to the image (Bitwise operations):


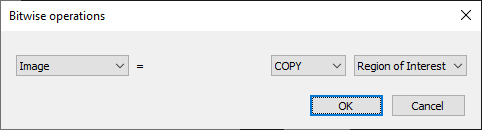


4.3.9.-Remove pores from the ROI (Despeckle 2D):


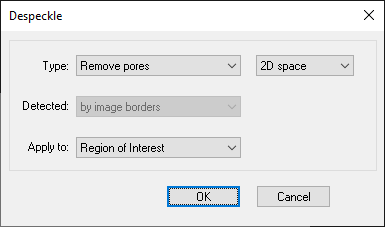


4.3.10.-Erode ROI (Morphological operations 2D):


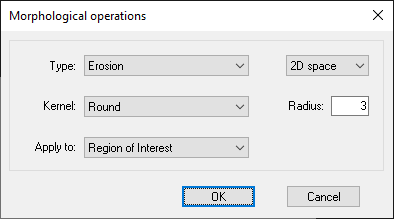


4.3.11.- Make a new image dataset fitting the ROI (Bitwise operations):


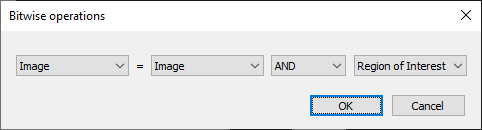


4.3.12.- Remove the outer non desired structures of the images stored into the clipboard (Bitwise operations):

Compare this resulting image with the one stored in the clipboard shown in step 3.3.3


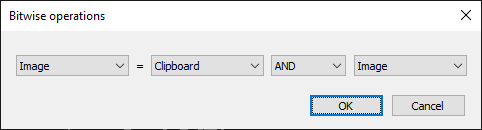

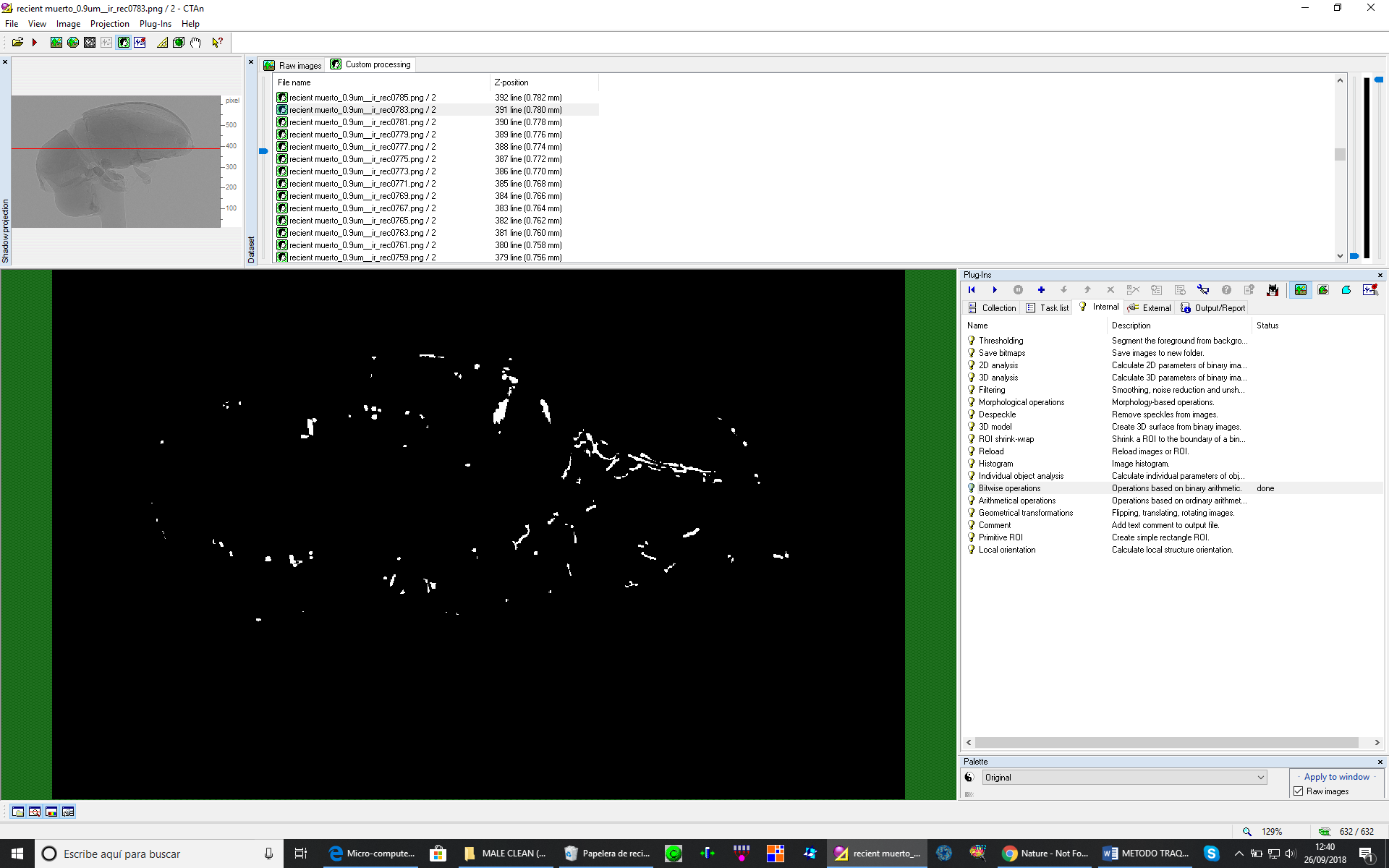


**5.- SAVE “CLEANED” PRELIMINARY FINAL IMAGE DATASET** (Save bitmaps):


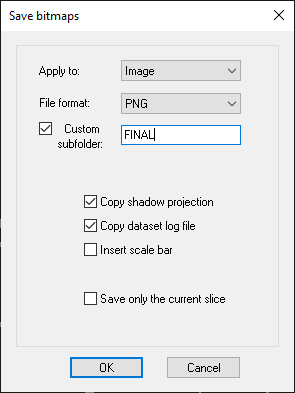

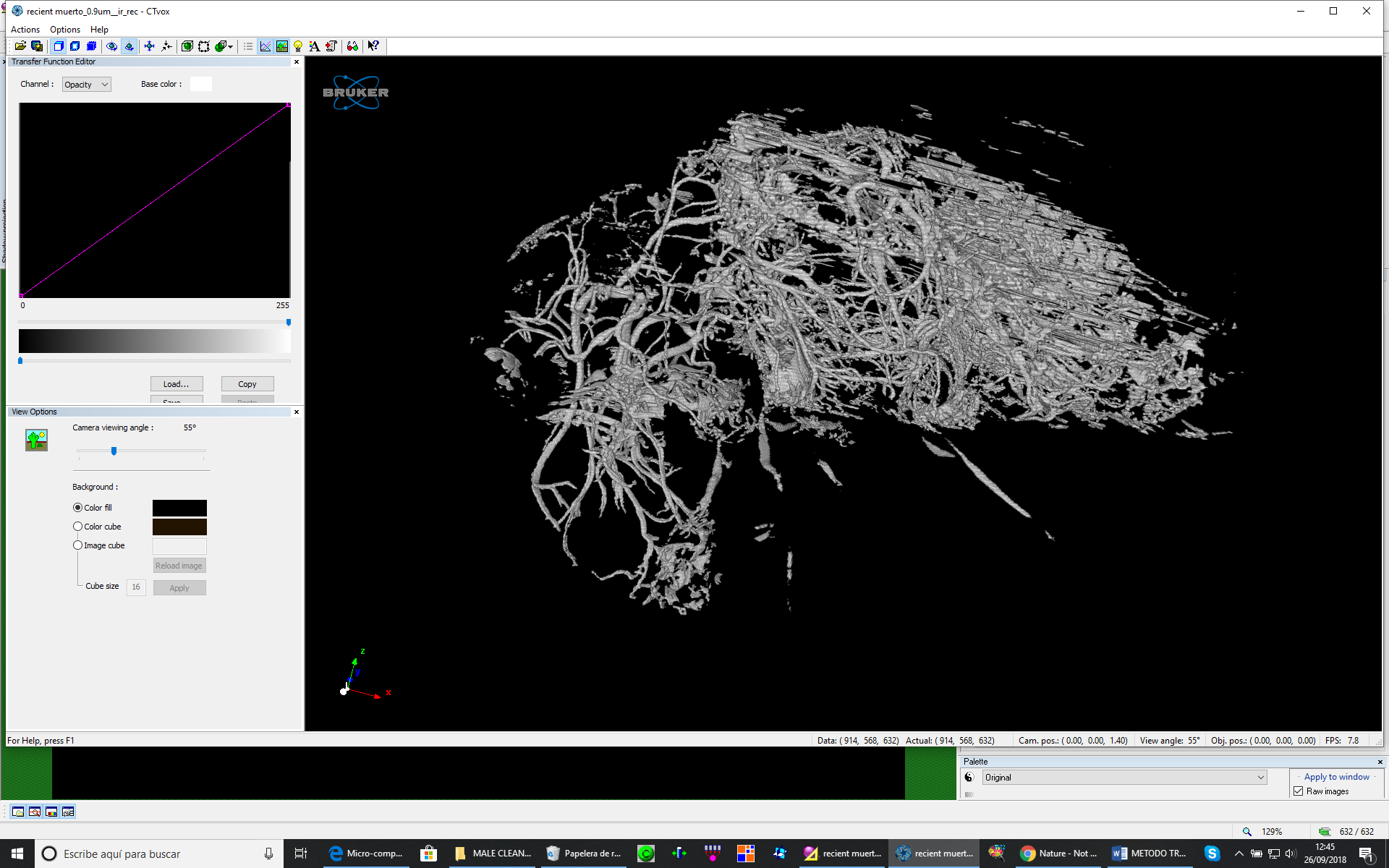


**6.- FINAL CLEANING OF THE IMAGES**

5.1 The dataset is uploaded into the Amira software, and by using the cutting (inside) tool of the “Volume Edit” module, and manually progressively removing the non- tracheal structures of the volume rendering, until a final clean volume rendering is obtained:


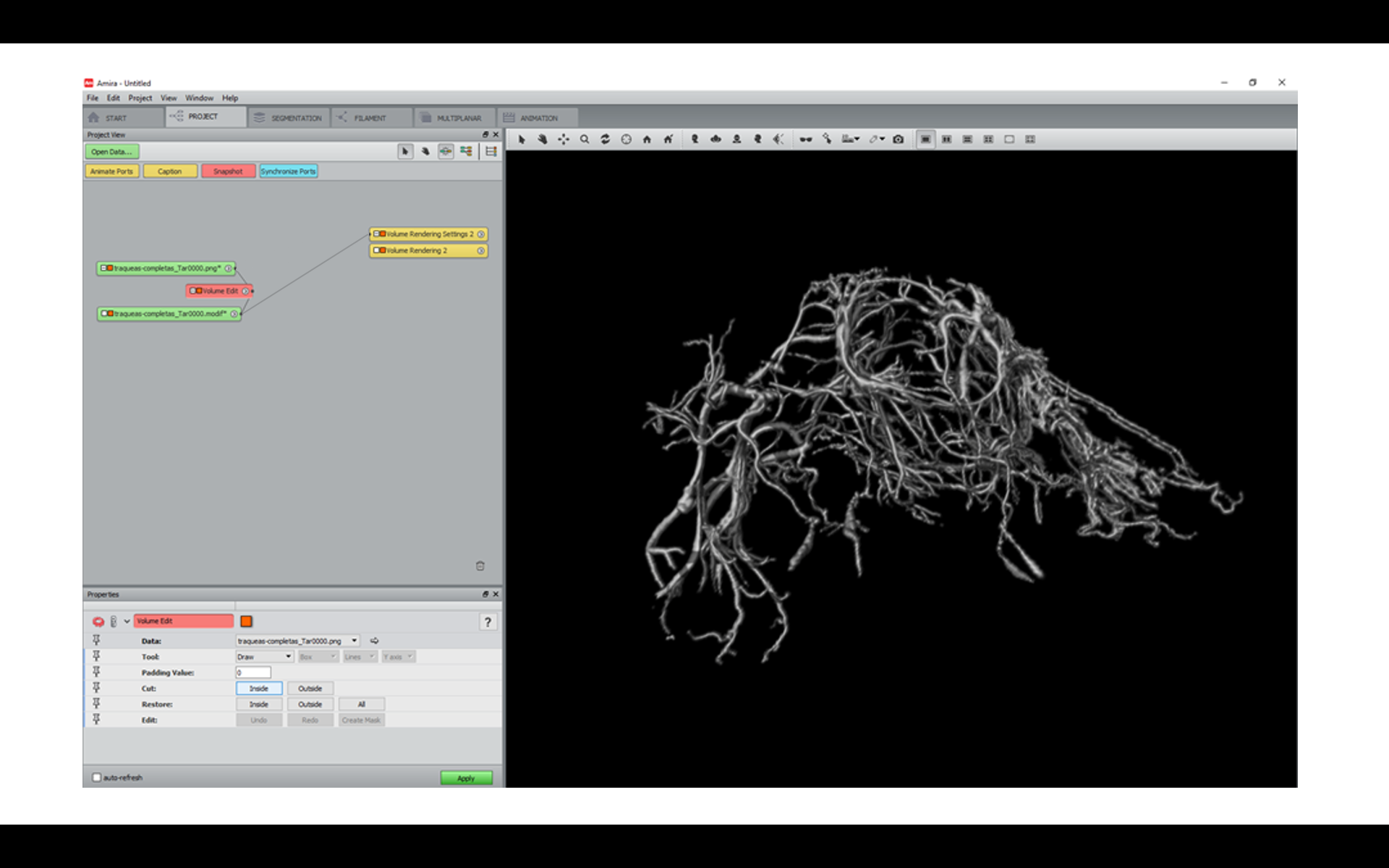


5.2.- Export the cleaned final image dataset (Export Data As):


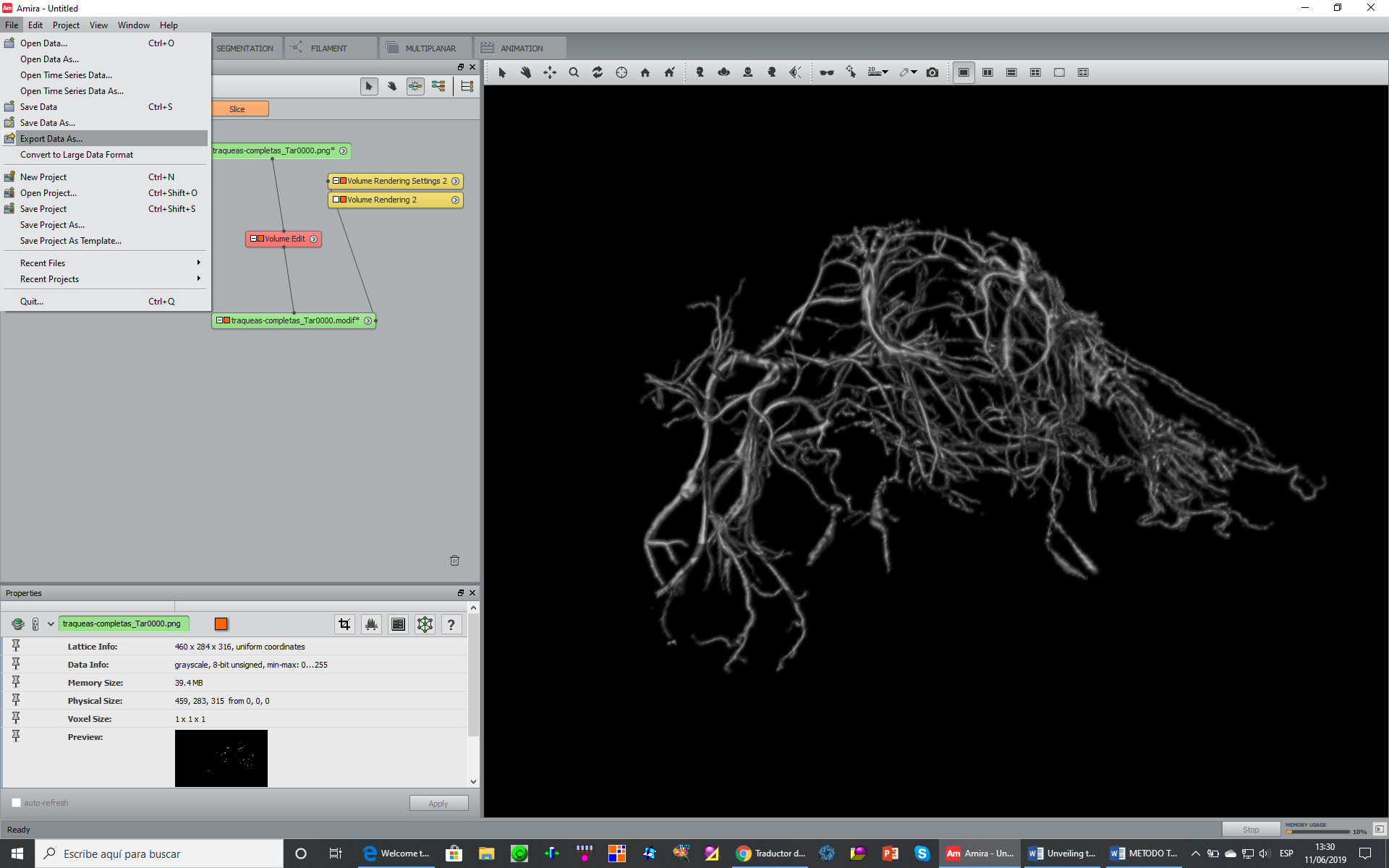


**Supplementary Videos (mp4)**

**Supplementary Video S1**

Tracheal system, Part I: an adult coffee berry borer female was progressively made transparent, thus allowing us to see the tracheal respiratory system and the complexity of tubes. External spinning views and a virtual tour inside the body are shown. There are two separate views: (1) the locations of the tracheal tubular system inside the body wall of the insect and (2) how this system supplies the different internal structures. Lumen size of the tracheal tubes is indicated in a color scale shown on the upper right corner.

**Supplementary Video S2**

Tracheal system, Part II: an adult coffee berry borer female was progressively made transparent, thus allowing us to see the tracheal respiratory system and the complexity of tubes. Lumen size of the tracheal tubes is indicated in a color scale shown on the upper right corner.

**Supplementary Video S3**

Tracheal system. Part III. As Supplementary Video S2 but showing details of the position of the respiratory spiracles.

**Supplementary Video S4**

Close-up view of the metathoracic spiracle structure and its connecting tracheal tubes. To facilitate differentiation of the tracheae, in some parts of the video the tracheal lumina was reconstructed in a red colour.

**Supplementary 3D model to visualise with mobile devices (vxm)**:

**Supplementary 3D model S5.** Tracheal tubular system and the teguments to be visualized with the CTvox app for mobile devices (smartphones and tablets, either with iOS or Android systems). To install CTvox on your device, visit Apple’s App Store or Google’s Play Store. The app is free of charge. Instructions can be downloaded by clicking on the following links:

1. For iOS devices:

a) iPad:

<https://www.bruker.com/fileadmin/user_upload/8-PDF-Docs/Microtomography/CTvoxForIpad.pdf>

b) iPhone:

<https://www.bruker.com/fileadmin/user_upload/8-PDF-Docs/Microtomography/CTvoxForIPhone.pdf>

2. For Android devices:

<https://www.bruker.com/fileadmin/user_upload/8-PDF-Docs/Microtomography/CTvoxForAndroid.pdf>
